# MR imaging of ovarian masses: classification and differential diagnosis

**DOI:** 10.1007/s13244-015-0455-4

**Published:** 2015-12-16

**Authors:** Pietro Valerio Foti, Giancarlo Attinà, Saveria Spadola, Rosario Caltabiano, Renato Farina, Stefano Palmucci, Giuseppe Zarbo, Rosario Zarbo, Maria D’Arrigo, Pietro Milone, Giovanni Carlo Ettorre

**Affiliations:** Radiodiagnostic and Radiotherapy Unit, University Hospital “Policlinico-Vittorio Emanuele”, Via Santa Sofia 78, 95123 Catania, Italy; Department G.F. Ingrassia – Institute of Pathology, University of Catania, Catania, Italy; Department of General Surgery and Medical-Surgical Specialties – Institute of Obstetrics and Ginecology, University of Catania, Catania, Italy; Pathology Unit, University Hospital “Policlinico-Vittorio Emanuele”, Catania, Italy

**Keywords:** Ovary, Magnetic resonance imaging, TNM staging, Ovarian cancer, Tumour staging

## Abstract

**Objective:**

We propose a Magnetic Resonance Imaging (MRI) guided approach to differential diagnosis of ovarian tumours based on morphological appearance.

**Background:**

Characterization of ovarian lesions is of great importance in order to plan adequate therapeutic procedures, and may influence patient’s management. Optimal assessment of adnexal masses requires a multidisciplinary approach, based on physical examination, laboratory tests and imaging techniques. Primary ovarian tumours can be classified into three main categories according to tumour origin: epithelial, germ cell and sex cord-stromal tumours. Ovarian neoplasms may be benign, borderline or malignant. Using an imaging-guided approach based on morphological appearance, we classified adnexal masses into four main groups: unilocular cyst, multilocular cyst, cystic and solid, predominantly solid. We describe MR signal intensity features and enhancement behaviour of ovarian lesions using pathologically proven examples from our institution.

**Conclusion:**

MRI is an essential problem-solving tool to determine the site of origin of a pelvic mass, to characterize an adnexal mass, and to detect local invasion. The main advantages of MRI are the high contrast resolution and lack of ionizing radiation exposure. Although different pathological conditions may show similar radiologic manifestations, radiologists should be aware of MRI features of ovarian lesions that may orientate differential diagnosis.

**Teaching Points:**

• *Diagnostic imaging plays a crucial role in detection, characterization and staging of adnexal masses.*

• *Characterization of an ovarian lesion may influence patient’s management.*

• *Different pathological conditions may have similar radiologic manifestations.*

• *Non-neoplastic lesions should always be taken into consideration.*

## Introduction

Ovarian masses are a common finding in daily clinical practice and may be incidentally detected or identified in symptomatic patients. Characterization of an ovarian lesion represents a diagnostic challenge; it is of great importance in the preoperative setting in order to plan adequate therapeutic procedures and may influence patient’s management.

Magnetic resonance (MR) imaging may provide useful information for the characterization of ovarian masses as non-neoplastic or neoplastic, and, in the latter case, as benign or malignant. Therefore, radiologists play an important role in the multidisciplinary approach of ovarian mass, and, though different pathological conditions may have similar radiologic manifestations, they should be aware of MR imaging features of ovarian lesions that may orientate the differential diagnosis.

In this article, we review epidemiology, etiopathogenesis, classification, staging and diagnostic approaches to ovarian masses. We propose an MR imaging-guided approach to the differential diagnosis of ovarian tumours based on morphological appearance. We describe MR signal intensity features (e.g., haemorrhagic areas, elevated protein content, fat and collagenous tissue) and enhancement behaviour of each lesion using pathologically proven examples from our institution. Our MR protocol is also included.

### Epidemiology

Ovarian cancer is currently one of the most common forms of cancer among women worldwide, responsible for 3.6 % of all cases, with a mortality of 4.3 %. In Europe, it is the leading cause of cancer death among gynaecologic malignancies, ranking fifth in incidence (exceeded only by breast, colorectum, lung and corpus uteri) and sixth in mortality among all women’s cancer (exceeded by breast, colorectum, lung, pancreas and stomach) [1]. An important reason for the high mortality rates of this cancer is the late diagnosis. Many patients present in advanced stage, mostly because the disease is often asymptomatic or associated with nonspecific symptoms in the early stage. Incidental detection of adnexal mass is very common in clinical practice.

### Etiopathogenesis

Etiopathogenesis of ovarian tumours is not fully understood; however, it appears to be multifactorial. The leading risk factor is familiarity; namely, history of ovarian cancer in a first-degree relative. However, only 5–10 % of cases are related to hereditary syndromes: the main one is the breast-ovarian cancer syndrome, due to mutations in the BRCA1 and BRCA2 tumour suppressor genes. Approximately 90–95 % of cases are sporadic, with an increasing risk related to nulliparity, early menarche and late menopause, while pregnancy, lactation, early menopause and use of oral contraceptives appear to be protective factors [2]. Hereditary cases occur predominantly in premenopausal age, while the sporadic ones affect mostly older women.

### Classification

Primary ovarian tumours can be classified into three main categories according to tumour origin: epithelial, germ cell and sex cord-stromal tumours; ovaries are also affected by metastastic tumours (Table 1) [3]. Epithelial tumours account for approximately 85 % of ovarian malignancy: the most common type is serous carcinoma. Dermoid cyst (mature cystic teratoma) is the most common benign ovarian neoplasm. Sex cord-stromal neoplasms may produce hormones, both oestrogen and androgens, resulting in endocrinological symptoms. Ovarian neoplasms may be benign, borderline or malignant. In addition, several benign lesions should also be considered, namely functional and haemorrhagic cyst, as well as endometriomas.

**Table 1:** Histological classification of ovarian tumours adapted from WHO

Primary tumours (95 %)	Surface epithelial-stromal tumours (65 %)	serous	benign	cystadenoma, papillarycystadenoma adenofibroma/cystadenofibroma
			borderline	papillary cystic tumour, surface papillary tumour, adenofibroma/cystadenofibroma
			malignant	adenocarcinoma, surfacepapillary adenocarcinoma, adenocarcinomafibroma
		mucinous	benign	cystadenoma, adenofibroma/cystadenofibroma
			borderline	intestinal type, endocervical-like
			malignant	adenocarcinoma, adenocarcinomafibroma
		endometrioid		
		clear cell		
		transitional cell (Brenner)		
		undifferentiated and unclassified		
	Germ cell tumours (15 %)	teratoma	biphasic or triphasic	mature
				immature
			monodermal	strumaovarii
		dysgerminoma		
		Yolk sac tumour		
		choriocarcinoma		
		embrionalcell carcinoma		
	Sex cord-stromal (10 %)	granulosa cell	adult	
			juvenile	
		thecoma-fibroma group	fibroma	
			techoma	
			sclerosing stromal tumour	
			unclassified (fibrothecoma)	
		Sertoli-Leydigcell		
		steroidcell		
	Miscellaneous (5 %)	small cell carcinoma, gestational choriocarcinoma, others		
Secondary tumours (5 %)		stomach, colon, breast, lung, contralateral ovary		

### Staging

There are two staging systems to describe the spread of ovarian tumours: the TNM (tumour, node, metastasis) and the International Federation of Gynecology and Obstetrics (FIGO). The FIGO system has been recently revised and went into effect on 1 January 2014 (Table 2). Basically, stage I describes tumours confined to ovaries, stage II reflects pelvic extension or primary peritoneal cancer, stage III indicates spread to the peritoneum outside the pelvis and/or metastasis to the retroperitoneal lymph nodes, while stage IV refers to distant metastasis. Ovarian cancer may show direct extension to surrounding pelvic structures, as well as intraperitoneal, lymphatic and hematogenous spread. Local invasion of uterus, fallopian tubes and contralateral adnexa can be found, while the involvement of bladder and rectum is less frequent. One of the most important features of ovarian cancer is intraperitoneal dissemination by exfoliation of cells, often associated with a variable amount of ascites: pouch of Douglas, greater omentum and subphrenic region are the most common sites of implantation, probably because of the preferential circulatory path of peritoneal fluid. Lymphatic dissemination is most common to para-aortic and paracaval lymph nodes, following the ovarian veins; pathologic lymph nodes can also be found in pelvic (external iliac, hypogastric and obturatory) and inguinal chains. Incidence of lymph node metastasis has been estimated to be about 25 % in stage I patients, about 50 % in patients with stage II disease and about 74 % in advanced stages. In addition, in patients with intraperitoneal disseminated disease, there is a possibility of approximately 30 % of pelvic, para-aortic or inguinal lymph nodes involvement [4]. Hematogenous metastases are less frequent and most often tend to involve liver and lung; however spleen, bone, brain and other locations may be affected.

**Table 2:** FIGO system for ovarian cancer staging

Stage I	Tumour confined to ovaries	
I A	Tumour limited to 1 ovary, capsule intact, no tumour on surface, negative washings	
I B	Tumour involves both ovaries otherwise like IA	
I C	Tumour limited to 1 or both ovaries	
	*I C1*	Surgical spill
	*I C2*	Capsule rupture before surgery or tumour on ovarian surface
	*I C3*	Malignant cells in the ascites or peritoneal washings
Stage II	Tumour involves 1 or both ovaries with pelvic extension (below the pelvic brim) or primary peritoneal cancer	
II A	Extension and/or implant on uterus and/or Fallopian tubes	
II B	Extension to other pelvic intraperitoneal tissues	
Stage III	Tumour involves one or both ovaries with cytologically or histologically confirmed spread to the peritoneum outside the pelvis and/or metastasis to the retroperitoneal lymph nodes	
III A	Positive retroperitoneal lymph nodes and /or microscopic metastasis beyond the pelvis	
	*III A1*	Positive retroperitoneal lymph nodes only
	*III A2*	Microscopic, extrapelvic (above the brim) peritoneal involvement ± positive retroperitoneal lymph nodes
III B	Macroscopic, extrapelvic, peritoneal metastasis ≤2 cm ± positive retroperitoneal lymph nodes. Includes extension to capsule of liver/spleen	
III C	Macroscopic, extrapelvic, peritoneal metastasis >2 cm ± positive retroperitoneal lymph nodes. Includes extension to capsule of liver/spleen	
Stage IV	Distant metastasis excluding peritoneal metastasis	
IV A	Pleural effusion with positive cytology	
IV B	Hepatic and/or splenic parenchymal metastasis, metastasis to extraabdominal organs (including inguinal lymph nodes and lymph nodes outside of the abdominal cavity)	

## Approach to adnexal masses

The optimal assessment of an adnexal mass requires a multidisciplinary approach, based on physical examination, laboratory tests and imaging techniques. An important issue to consider in the management of ovarian masses is that they are very common, but most of them are benign and only a small part is borderline or malignant. Preoperative biopsy should not be performed in ovarian masses, particularly if the mass appears to be surgically resectable at the moment, as this invasive procedure raises the risk of spreading cancer cells and potentially leads to iatrogenic upstaging worsening the prognosis.

### Serum markers

The most commonly used serum tumour marker for epithelial tumours is cancer antigen (CA)-125. In postmenopausal women, CA-125 has high sensitivity as well as high specificity, while in premenopausal women, it has an high sensitivity but low specificity, because it may also be elevated in other conditions (e.g., peritoneal inflammation, endometriosis, etc.). Human epididymis protein 4 (HE4) is a recently added tumour marker and several studies have shown his usefulness in discriminating between benign and malignant adnexal masses in premenopausal women [5, 6].

### Diagnostic imaging

Diagnostic imaging plays a crucial role in detection, characterization and staging of adnexal masses.

*Ultrasound (US)* is often the first imaging study performed in the evaluation of a suspected ovarian lesion because it’s widely available, well accepted by patients, non-invasive and of low cost. A combination of greyscale and colour Doppler features, obtained with transabdominal and/or endovaginal scanning, allow for investigation of both morphological structure and vascular organization of the ovarian mass. Morphological features suggestive of malignancy include thickness (>2–3 mm) and irregularity of walls and septa, the presence of solid areas and papillary projections, as well as other evidence of malignant activity, namely ascites, peritoneal nodules and metastatic lesions. Regarding the vascularization, colour Doppler study is able to demonstrate both the presence and the localization of new tumour blood vessel: a predominantly central blood flow is more often associated with malignancy, while a peripheral one is more typical of a benign lesion [7]. In an ultrasound-indeterminate adnexal mass, Doppler ultrasound has shown a sensitivity of 84 % and a specificity of 82 % in diagnosing cancer [8]. Wu et al., on a recent meta-analysis of ten independent studies, reported a high diagnostic accuracy of contrast-enhanced ultrasound in distinguishing between benign and malignant ovarian masses [9]. However, unless morphological and vascularity features clearly indicate a benign lesion, further assessment is mandatory. Levine et al., in a consensus statement (2009) for the Society of Radiologists in Ultrasound, made recommendations about management of adnexal masses: “Adnexal masses in the physiologic range in terms of size and appearance in a woman of menstrual age or a simple adnexal cyst less than or equal to 1 cm in a postmenopausal woman are likely benign; these findings are almost always of no clinical importance in asymptomatic women and can be safely ignored” [10].

*Computed Tomography (CT)* of the abdomen and pelvis after contrast administration is important both in evaluation of spread of malignant lesions and in detection of recurrence after therapy, whereas it has a limited value in primary detection and characterization of an ovarian mass. With CT scans, only lesions containing fat tissue and calcifications, like mature teratoma, can be easily characterized. In a sonographically indeterminate adnexal mass, CT has shown a sensitivity of 81 % and a specificity of 87 % in ovarian cancer diagnosis [8]. CT is the imaging technique of choice in staging: looking for omental and peritoneal implants, ascites and lymphadenopathy is very important to assess the extension of the disease [8]. Therapy response evaluation is usually performed with CT, comparing pre-treatment with post-treatment scans (preferably after six cycles of chemotherapy). An interval between the CT scans of only three cycles of chemotherapy is indicated if serum markers are negative or their levels are not decreasing [11].

^*18*^*F-FDG PET/CT* is being increasingly used and its role in the evaluation of ovarian tumours appears to be crucial in the postoperative follow-up of patients with suspected recurrence [8, 12]. This imaging modality demonstrated a sensitivity of up to 91 % and a specificity up to 100 % in the detection of cancer recurrence, and plays a crucial role especially when CT scans are negative but serum marker levels increase [11]. PET/CT is not usually performed in the initial evaluation of these patients, mostly because it may lead both to false-positive and false-negative results. It has to be considered that several benign lesions, particularly teratomas and endometriomas, may show FDG uptake, whereas small (<1 cm), necrotic and low-grade tumour may not [4]. However, finding an increased FDG uptake in postmenopausal women has always to be considered an abnormality.

*Magnetic Resonance Imaging (MRI)* is an essential problem solving tool to determine the site of origin of a pelvic mass and then to characterize an adnexal mass, especially in patients with indeterminate lesions [13, 14]. MRI is also reliable in detecting local invasion. The main advantages of MRI are the high contrast resolution with excellent soft tissue contrast and lack of ionizing radiation exposure, which is particularly important in young female patients.

In order to obtain anatomic information and to study morphological and signal intensity characteristics of the mass, both T1- and T2-weighted sequences are needed. Fat-saturated T1-weighted images are helpful to detect haemorrhagic areas and fat tissue. The use of intravenous gadolinium improves detection of enhancing septa and solid components within the mass and of peritoneal and omental implants.

In the evaluation of adnexal masses indeterminate on ultrasound, unenhanced MRI has shown a sensitivity and a specificity of 76 and 97 %, respectively, in the diagnosis of ovarian cancer; assessment with contrast-enhanced MRI increases sensitivity to 81 % and specificity to 98 % [8].

Diffusion-weighted imaging (DWI) is a potentially useful technique in the assessment of adnexal masses; however, its role has been controversial in literature. For Katayama et al. [15] and Fujii et al., [16], DWI respectively “is not useful” and “provide no additional information” in discriminating benign from malignant ovarian masses. In 2009, Thomassin-Naggara et al. demonstrated that the combination of diffusion-weighted and T2-weighted images is helpful in predicting benignity and malignancy: masses with low signal intensity on both sequences were more likely benign, while lesions with high signal intensity on DWI and intermediate signal on T2-weighted images were more likely malignant [17]. Recently, other studies [18, 19] have shown that high signal intensity on DWI is more frequent within malignant lesions and is useful for differentiating them from benign ones. It has to be considered that several benign lesions, namely endometriomas, teratomas and fibrothecomas, may also show restricted diffusion; however, a confident diagnosis of these lesions can usually be done with T1-weighted, T1-weighted fat-suppressed and T2-weighted standard sequences [20]. In a paper published in 2010, Kyriazi et al. investigated the role of DWI in the imaging of peritoneal carcinomatosis in patients with advanced ovarian cancer, showing that it could be helpful in the assessment of volume and location of disease sites, as well as in monitoring recurrence [21]. In 2014, Zhao et al. demonstrated that DWI is useful in the differentiation of borderline and malignant epithelial ovarian tumours [22]. In our opinion, diffusion-weighted images should be included in MRI protocol.

The role of 1.5 T MRI in the assessment of ovarian masses has been widely established, but only in recent years have 3 T MR systems been applied in the study of gynaecologic diseases.

As for the magnetic field strength, the main advantage of 3 T MR systems is the expected increase in MR signal-to-noise ratio (SNR) of up to twofold as compared with standard 1.5 T MR scanners; this gain in SNR can be used to improve either speed or spatial resolution, or both. Nevertheless pulse sequence parameters at 3 T need to be reoptimized from 1.5 T values in order to maintain desired image contrast. Image artefacts due to changes in tissue susceptibility, chemical shift, radiofrequency effects, and/or pulse sequence physics may be more noticeable and harder to suppress at 3 T [23]. In particular, DWI sequences can suffer from increased warping and susceptibility artefacts at 3 T [23]. Previous studies [24] have shown that when body DWMRI protocols are transferred from 1.5 to 3 T without further modifications, image quality is seriously degraded and images suffer from severe distortions and signal losses, so qualitative assessment and quantitative analysis can be problematic. Therefore, protocol adjustment at 3 T is mandatory because compromised image quality may have serious implications in diagnosis.

Proton MR spectroscopy is a non-invasive diagnostic tool that may contribute to the differential diagnosis of subtypes in ovarian tumours. In addition to the 1.5 T system, the superior spectral separation and increased signal-to-noise ratio offered by 3 T systems allow high quality MR spectroscopy. The various subtypes of malignant epithelial ovarian tumours (serous, clear cell, endometrioid, and mucinous) respond differently to chemotherapy. In particular, serous adenocarcinoma may have a good response to chemotherapy, whereas clear cell and mucinous adenocarcinomas may show poor response to chemotherapy. Proton MR spectroscopy may identify the presence of mucinous material containing N-acetyl mucinous compounds, and can provide helpful information in distinguishing mucinous and nonmucinous ovarian tumours. Therefore, MR spectroscopy helps to diagnose the subtypes of ovarian tumours and may contribute to the adequate treatment, thus improving management of these patients [25].

Compared with 1.5 T, the increased signal-to-noise ratio and improved background suppression at 3 T may allow better categorization of variable components (fluid, blood, lipid, etc.). In addition, in the study of complex adnexal masses, dynamic contrast-enhanced MRI is especially effective for increasing the conspicuity of findings that are predictive of malignancy. Owing to its higher diagnostic performance, 3 T MRIs categorize and characterize the primary adnexal lesions well, and could be regarded as a reliable non-invasive modality method for distinguishing malignancies from benign tumours, though histological examination is needed to determine the final diagnosis [26].

## Our MR protocol

The MR imaging protocol we use in our institution to study patients with ovarian masses is as follows.

MR imaging is performed with a closed-configuration superconducting 1.5-T system (Signa HDxT; GE Healthcare, Milwaukee, Wis) with 57.2 mT/m gradient strength and 120 T/m/s slew rate, by using an eight-channel high-resolution torso coil with array spatial sensitivity technique (ASSET) parallel acquisition.

MR sequences

- Localizer sequence in the three spatial planes;
- axial T2-weighted single-shot fast spin-echo (SSFSE) sequence [time to repetition (TR)/time to echo (TE) range 765/59; flip angle 90°; section thickness 6 mm; interslice gap 0.6 mm; bandwidth 31.25 kHz; field of view (FOV) 38 cm; matrix 320 × 288; number of averages 0.54; number of images 30; acquisition time 24 s] used as second localiser to identify the longitudinal axis of the uterus in the case of laterally deviated uterus;
- sagittal T2-weighted fast spin-echo (FSE) sequence parallel to the longitudinal axis of the uterus (identified on the previous SSFSE sequence) (TR/TE range 4675/100; flip angle 90°; section thickness 4 mm; interslice gap 1 mm; bandwidth 41.67 kHz; FOV 32 cm; matrix 320 × 224; number of averages 4; number of images 26; acquisition time 3 min 49 s);
- oblique coronal T2-weighted FSE sequence parallel to the longitudinal axis of the uterus (TR/TE range 4675/100; flip angle 90°; section thickness 4 mm; interslice gap 1 mm; bandwidth 41.67 kHz; FOV 32 cm; matrix 320 × 224; number of averages 4; number of images 26; acquisition time 3 min 49 s);
- oblique axial T2-weighted FSE sequence perpendicular to the longitudinal axis of the uterus (TR/TE range 4675/100; flip angle 90°; section thickness 4 mm; interslice gap 1 mm; bandwidth 41.67 kHz; FOV 32 cm; matrix 320 × 224; number of averages 4; number of images 26; acquisition time 3 min 49 s);
- sagittal or axial oblique or coronal oblique fat suppressed T2-weighted FSE sequence (TR/TE range 4675/100; flip angle 90°; section thickness 4 mm; interslice gap 1 mm; bandwidth 41.67 kHz; FOV 32 cm; matrix 320 × 224; number of averages 4; number of images 24; acquisition time 3 min 49 s);
- axial T1-weighted gradient-echo (GRE) sequence in-out (chemical-shift imaging) (TR/TE 180/2,1; flip angle 80°; section thickness 6 mm; interslice gap 0,6 mm; bandwidth 62,5 kHz; field of view 38 cm; matrix 256 × 224; number of averages 1; number of images 20; acquisition time 22 s);
- axial DWI SE EPI (TR/TE 3000/74,1; flip angle 90°; section thickness 5 mm; interslice gap 1 mm; bandwidth 250 kHz; field of view 45 cm; matrix 160 × 160; number of averages 16; number of images 14; b-value 0 e 800 s/mm^2^; acquisition time 1 min e 40 s);
- sagittal, coronal oblique, axial oblique T1-weighted 3D gradient-echo liver acquisition with volume acquisition (LAVA) sequence with fat suppression (TRe/TE range 4.4/2.1; flip angle 12°; section thickness 3.4 mm; overlap locations −1.7 mm; bandwidth 62.5 kHz; FOV 40 cm; matrix 320 × 192; number of averages 0.75; number of images 104; acquisition time 22 s).

After i.v. administration of 0.1 mmol/kg paramagnetic contrast agent (Dotarem, Guerbet, Roissy, France) at a flow rate of 2 ml/s, followed by 20 ml of saline solution at the same flow rate, the following sequences are acquired:

- dynamic sagittal T1-weighted 3D gradient-echo LAVA with fat suppression (TR/TE range 4.4/2.1; flip angle 12°; section thickness 3.4 mm; overlap locations −1.7 mm; bandwidth 62.5 kHz; FOV 40 cm; matrix 320 × 192; number of averages 0.75; number of images 104; acquisition time 22 s) acquired at 60 and 120 s after contrast administration;
- T1-weighted 3D gradient echo LAVA fat-suppressed sequence, in the coronal oblique (parallel to the longitudinal axis of the uterus), axial oblique (perpendicular to the longitudinal axis of the uterus) and axial planes (TR/TE range 4.4/2.1; flip angle 12°; section thickness 3.4 mm; overlap locations −1.7 mm; bandwidth 62.5 kHz; FOV 40 cm; matrix 320 × 192; number of averages 0.75; number of images 104; acquisition time 22 s).

MR imaging is performed with the patient lying in the supine position (feet first). T2-weighted FSE sequences and DW sequences are acquired with patient breathing freely, T1-weighted 3D gradient echo LAVA fat-suppressed sequence are acquired in breath hold.

In case of voluminous ovarian masses that exceed the FOV of the above-mentioned sequences, we use the following MR protocol.

- Standard three-plane scout image;
- coronal T2-weighted SSFSE with and without fat suppression (TR range/TE range 705/90; flip angle 90°; section thickness 6 mm; interslice gap 0.6 mm; bandwidth 83.33 kHz; field of view 44–48 cm; matrix 384 × 224; number of signals acquired 0.57; number of images 28; acquisition time 24 s);
- axial T2-weighted SSFSE with and without fat suppression [time to repetition (TR)/time to echo (TE) range 765/59; flip angle 90°; section thickness 6 mm; interslice gap 0.6 mm; bandwidth 31.25 kHz; field of view (FOV) 38 cm; matrix 320 × 288; number of averages 0.54; number of images 30; acquisition time 24 s];
- fast imaging employing steady-state acquisition (FIESTA) in the coronal, axial and sagittal planes (TR range/TE range 4/1.7; flip angle 75°; section thickness 6 mm; interslice gap 0.6 mm; bandwidth 100 kHz; field of view 44–48 cm; matrix 320 × 224; number of signals acquired 1; number of images 28; acquisition time 22 s);
- axial DWI SE EPI [TR range/TE range 3000/74; flip angle 90°; section thickness 8 mm; interslice gap 2 mm; field of view 42 cm; matrix 160 × 160; number of signals acquired 2; number of images 15; b-value 0 and 800 s/mm^2^ that represent the best compromise between signal to noise ratio (SNR) and lesion detection sensitivity on our MR system; ASSET 2; acquisition time 27 s];
- sagittal, axial and coronal T1-weighted 3D gradient echo LAVA fat-suppressed sequence (TR range/TE range 4.1/1.9; flip angle 12°; section thickness 3.4 mm; overlap locs −1.7 mm; bandwidth 62.5 kHz; field of view 44–48 cm; matrix 320 × 192; number of signals acquired 0.70; number of images 120; acquisition time 23 s).

After i.v. administration of 0.1 mmol/kg paramagnetic contrast agent (Dotarem, Guerbet, Roissy, France) at a flow rate of 2 ml/s, followed by 20 ml of saline solution at the same flow rate, the following sequences are acquired:

- sagittal T1-weighted 3D gradient echo LAVA fat-suppressed sequence (TR range/TE range 4.1/1.9; flip angle 12°; section thickness 3.4 mm; overlap locs −1.7 mm; bandwidth 62.5 kHz; field of view 44–48 cm; matrix 320 × 192; number of signals acquired 0.70; number of images 120; acquisition time 23 s) acquired at 60 and 120 s after contrast administration;
- axial T1-weighted 3D gradient echo LAVA fat-suppressed sequence (TR range/TE range 4.2/2; flip angle 12°; section thickness 3.4 mm; overlap locs −1.7 mm; bandwidth 62.5 kHz; FOV 40 cm; matrix 320 × 192; number of signals acquired 0.70; number of images 120; acquisition time 23 s);
- coronal T1-weighted 3D gradient echo LAVA fat-suppressed sequence (TR range/TE range 4.1/1.9; flip angle 12°; section thickness 3.4 mm; overlap locs −1.7 mm; bandwidth 62.5 kHz; field of view 44–48 cm; matrix 320 × 192; number of signals acquired 0.70; number of images 120; acquisition time 23 s).

MR imaging is performed with the patient lying in the supine position (feet first). All sequences are acquired in breath hold.

## Imaging findings

Using an imaging guided approach based on morphological appearance, we classified adnexal masses into four main groups (Table 3):

**Table 3:** Classification of adnexal masses based on morphological appearance

Classification of adnexal masses basing on morphological appearance		
Cystic unilocular *(benign)*	non ovarian	paraovarian cysts, hydrosalpinx, pyosalpinx and hematosalpinx
	ovarian	functional cysts and serous cystadenomas (common)
		cystadenofibromas and mucinous cystadenomas (less common)
Cystic multilocular *(benign and borderline)*	endometriomas, mucinous cystadenomas and borderline tumours (common)	
	serous cystadenomas (less common)	
Cystic and solid *(benign, borderline and malignant)*	benign	mature cystic teratoma
	borderline to malignant	surface epithelial tumours, metastasis, Yolk sac, granulosa cell and Sertoli-Leydig tumours
Predominantly solid *(benign, borderline and malignant)*	benign	Brenner tumour, fibrothecomas
	borderline to malignant	serous and mucinous carcinomas, dysgerminoma and Yolk sac tumour, granulosa and Sertoli-Leydig cell tumours, metastasis

- unilocular cyst;
- multilocular cyst;
- cystic and solid;
- predominantly solid.

Then we evaluated signal intensity features (e.g., haemorrhagic areas, elevated protein content, fat and collagenous tissue) and enhancement behaviour for each lesion (Table 4).

**Table 4:** Chart summarizes the typical imaging features of the different ovarian lesions

Group	Lesion	Findings	T2	T1	Gd-T1	Mean age
Cystic unilocular	Functional cysts	follicles (diameter <20 mm), dominant follicles (diameter 20–25 mm), follicular cysts, corpus luteum cysts.	high	low corpus luteum may show high signal	no enhancement corpus luteum may enhance	reproductive age
	Serous cystadenoma	often bilateral, thin regular wall (<3 mm) no internal septations, papillary projections or solid components	high	low	no enhancement	
	Cystadenofibroma	sometimes: purely cystic lesion more often: complex cystic appearance with thick septa and solid components	high fibrous stroma: low signal intensity	low	no enhancement	
Cystic multilocular	Endometriosis	haemorrhagic content	intermediate to low shading sign	high	no enhancement	reproductive age
	Mucinous cystadenoma	thin regular wall, several septations, no solid components monolateral	variable signal intensity (stained glass appearance)		no enhancement	
	Borderline tumours	septa, papillary projections	intermediate	intermediate	enhancement of septa and papillary projections	45 younger patients than malignant ovarian cancer
Cystic and solid	Mature cystic teratoma	complex, heterogeneous appearance fat-tissue content	fat-tissue: high fat-tissue: low on fat-saturated sequences teeth: low signal intensity		variable	35
	Struma ovarii (monodermal teratoma)	complex mass with cystic spaces of variable signal intensity and solid areas thyroid tissue: thyrotoxicosis	cystic spaces with both high and low signal intensity cystic spaces with low signal intensity because of the colloid of the struma		enhancement of the cystic wall and solid components	50
	Ovarian metastasis	more often bilateral with a cystic and solid or a predominant solid morphological appearance from stomach, colon, breast, lung, contralateral ovary	intermediate to high	low to intermediate	enhancement of the cystic wall and solid components	
	Serous cystadenocarcinoma	complex multilocular masses, thick and irregular walls, septations, solid components and papillary projections frequently bilateral	cystic: high solid: low	cystic: low to intermediate solid: intermediate	enhancement of walls, septations, solid components and papillary projections	60
	Mucinous cystadenocarcinoma	complex multilocular masses, thick and irregular walls, septations, solid components and papillary projections	cystic: high solid: low mucinous: variable	cystic: low to intermediate solid: intermediate mucinous: variable	enhancement of walls, septations, solid components and papillary projections	
	Endometrioid adenocarcinoma	complex masses with solid and cystic components associated with endometriosis	haemorrhagic areas: intermediate	haemorrhagic areas: high	enhancement of walls and solid components	50–60
	Yolk sac tumour	mixed cystic and solid mass	haemorrhagic areas: intermediate	haemorrhagic areas: high	bright dot sign: foci of enhancement, dilated vessels	15–25
	Granulosa cell tumours	mixed cystic and solid mass hyperestrogenism, endometrial hyperplasia	cystic: high haemorrhagic: high solid: intermediate	cystic: low haemorrhagic: high solid: intermediate	enhancement of walls and solid components	60
Predominantly solid	Brenner tumour	fibrous content, calcifications	low	low to intermediate	no enhancement	50–70
	Dysgerminoma	lobulated lesion with fibrovascular septa, surrounded by a fibrotic capsule	solid component: intermediate to high septa: low	low to intermediate	enhancement of solid components and septa	25
	Fibrothecomas	fibrous tissue theca cells with lipidic content	low to intermediate	low to intermediate	minimal enhancement	60
	Fibromas	prominent fibrosis with abundant collagen content	low	low	moderate enhancement	60
	Thecomas	mainly lipidic content of theca cells	intermediate	intermediate lipidic content: low at chemical-shift (out of phase)	minimal enhancement	60
	Sertoli-Leydig cell tumours	solid mass or mixed cystic and solid mass 1/3 patients: signs of androgen activity	solid component: low scattered cystic areas: high	solid component: intermediate	enhancement of solid components	25–30

### Unilocular cystic masses

Unilocular cystic masses in the adnexal region are more likely benign and can have both non-ovarian or ovarian origin. Paraovarian cysts, hydrosalpinx, pyosalpinx and hematosalpinx are the most common extraovarian lesions, whereas ovarian lesions are usually represented by functional cysts and serous cystadenomas; less common unilocular ovarian cystic masses are cystadenofibromas and mucinous cystadenomas (more often multilocular). Most unilocular cystic masses have low signal intensity on T1-weighted images and high signal intensity on T2-weighted images.

*Paraovarian cysts* (or paratubal cysts or hydatid cysts of Morgagni) arise from mesothelial, paramesonephric or mesonephric remnants. It is important to identify the ipsilateral ovary as a separate structure in order to avoid misinterpretation; however, sometimes a “beak sign” can be found [27].

*Hydrosalpinx* is a fluid filled fallopian tube; it may assume a cystic appearance on some scans mimicking an ovarian lesion, but multiplanar visualization is usually able to demonstrate its sausage-like C-shape or S-shape tubular structure. Fallopian tubes can also be distended by pus or blood, respectively *pyosalpinx* (high signal intensity on diffusion-weighted images) and *hematosalpinx* (high signal intensity on T1-weighted images) [28].

*Functional cysts* are the most commonly encountered cystic masses in women of reproductive age as a normal part of the menstrual cycle, including follicles (diameter <20 mm), dominant follicles (diameter 20–25 mm), follicular cysts (resulting from persistence of an unruptured follicle) and corpus luteum cysts (resulting from a failure of the corpus luteum to regress). The latter one may enlarge because of an internal bleeding, showing high signal on T1-weighted images: follow-up is able to differentiate haemorrhagic corpus luteum cysts from endometrioma considering complete resolution of the functional cyst.

**Fig. 1:**
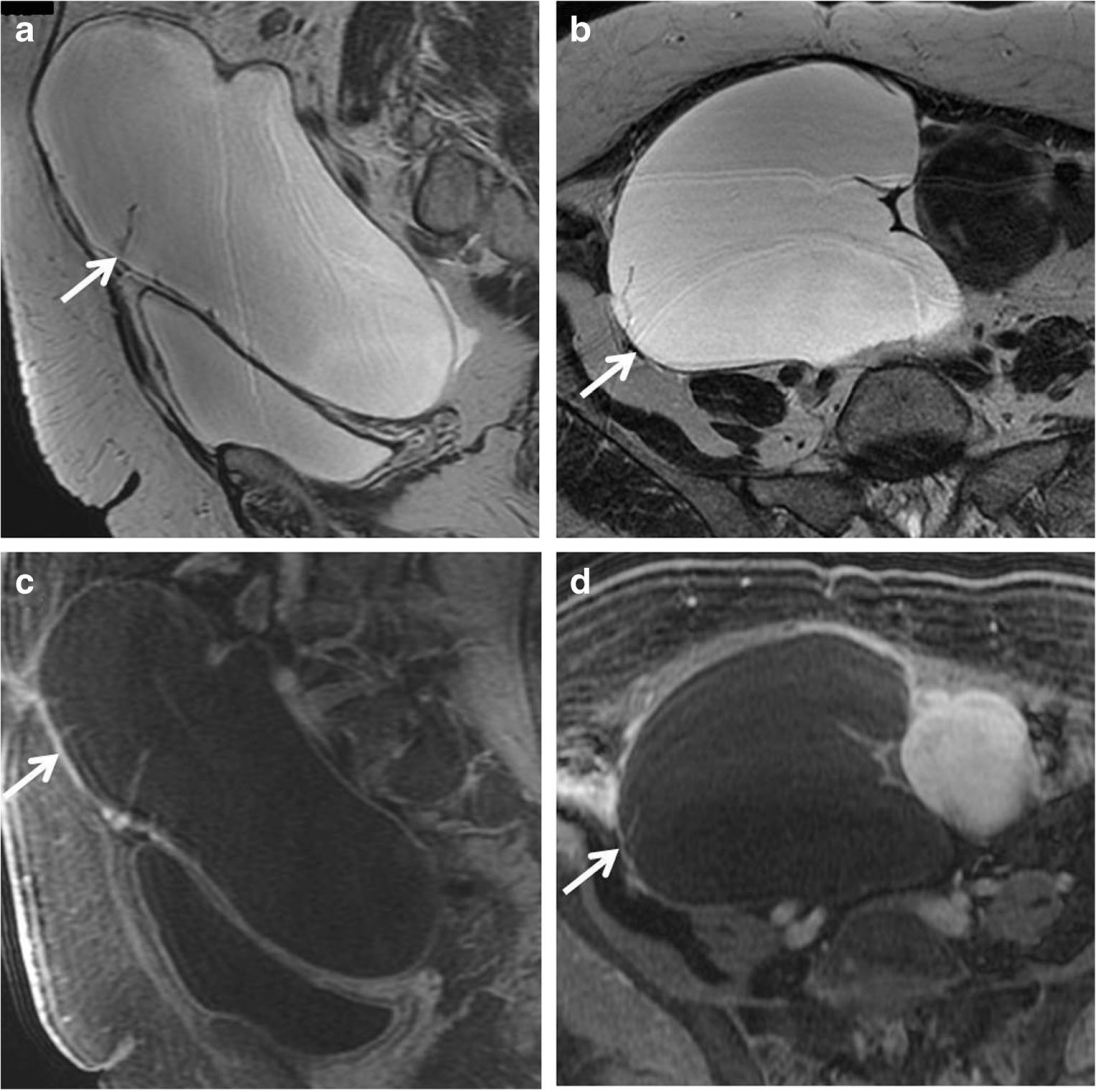
Serous cystadenoma in a 64-year-old woman. (**a**) Sagittal and (**b**) axial T2-weighted images show a hyperintense unilocular cystic mass (*white arrows*). On (**c**) sagittal and (**d**) axial contrast-enhanced fat-suppressed T1-weighted images, the cyst wall shows poor contrast enhancement (*white arrows*) without vegetations, nodularity, or solid components

*Serous cystadenoma* is a benign tumour that usually present as an unilocular cyst with a thin regular wall (less than 3 mm); the lining is flat without internal septations, papillary projections or solid components, showing no significative contrast enhancement. Serous fluid shows low signal intensity on T1-weighted and high signal intensity on T2-weighted images (Fig. 1). They are usually smaller and more often bilateral than mucinous cystadenomas. Serous cystadenomas of borderline malignancy may show some small papillary projections.

**Fig. 2:**
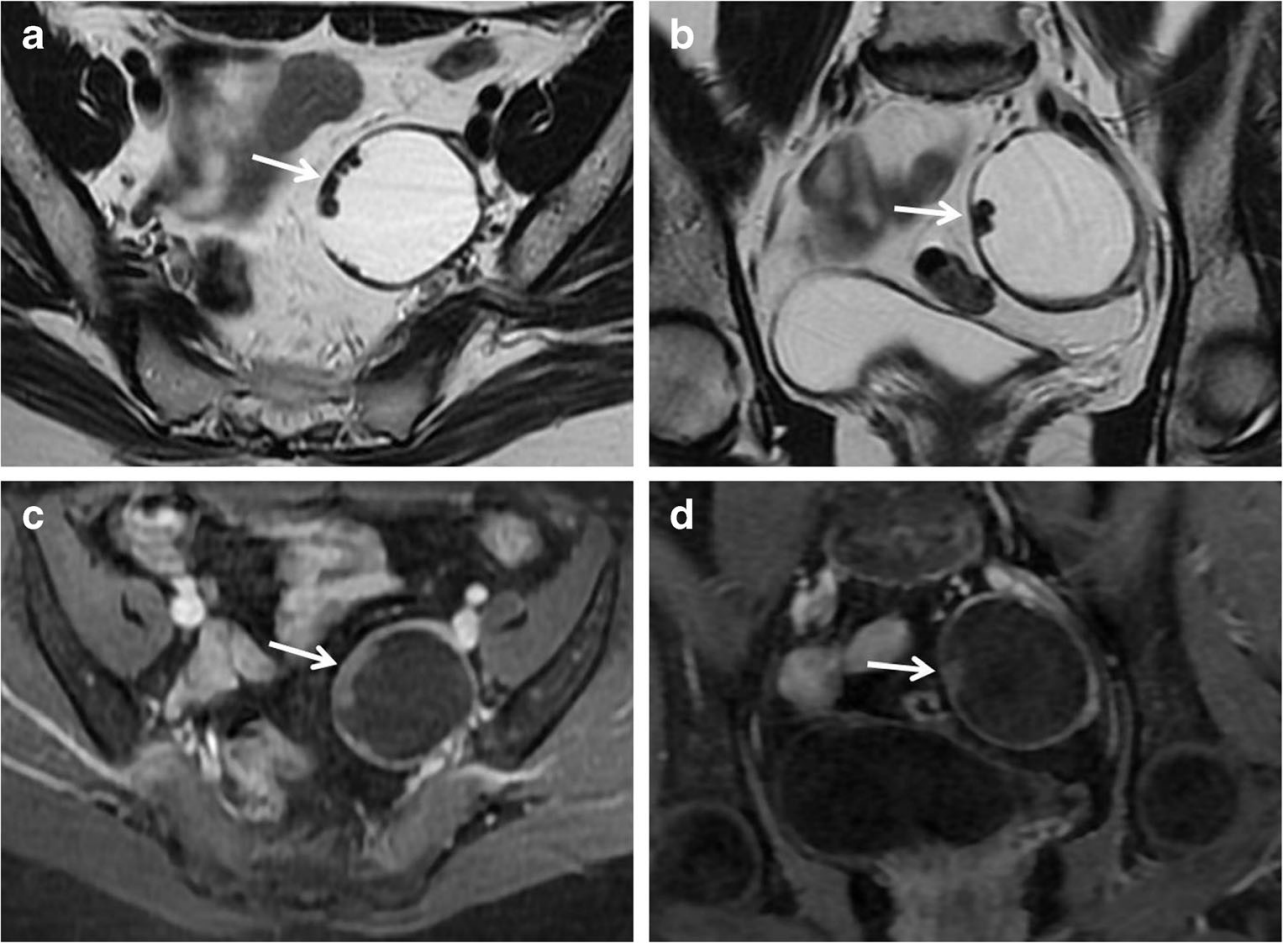
Cystadenofibroma in a 54-year-old woman. (**a**) Axial and (**b**) coronal T2-weighted images show a predominantly cystic mass with small areas of fibrous stroma along the wall characterized by homogeneous low signal intensity (*white arrows*). (**c**) Axial and (**d**) coronal contrast-enhanced fat-suppressed T1-weighted images show minimum enhancement of the cystic wall and of the fibrous component (*white arrows*)

**Fig. 3:**
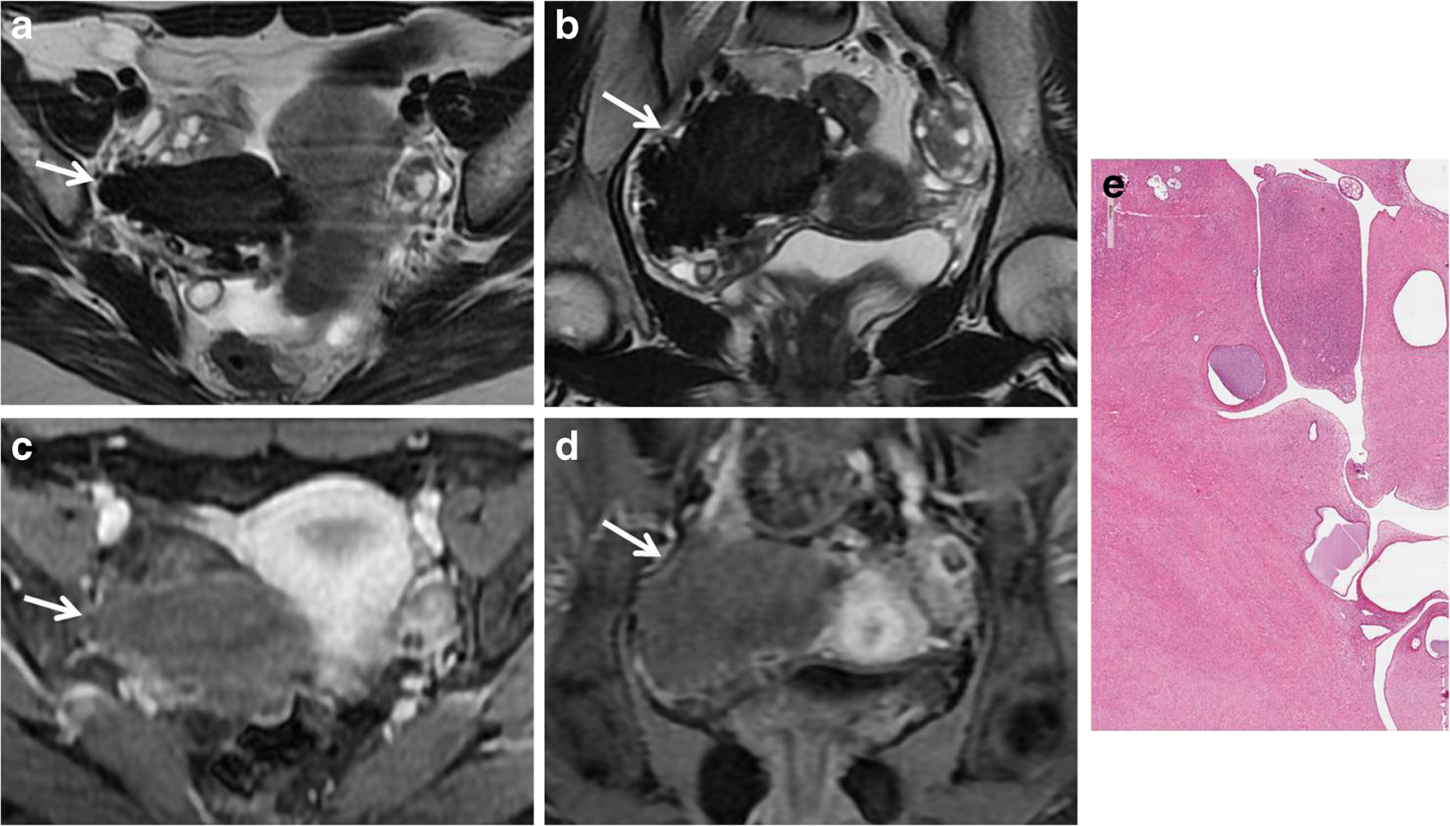
Cystadenofibroma in a 29-year-old woman. (**a**) Axial and (**b**) coronal T2-weighted images show a predominantly solid mass with prevalence of the fibrous stroma characterized by homogeneous low signal intensity (*white arrows*). (**c**) Axial and (**d**) coronal contrast-enhanced fat-suppressed T1-weighted images demonstrate minimum enhancement of the lesion (*white arrows*). (**e**) Photomicrograph (H&E X30) shows that the epithelial elements are surrounded by abundant fibrous stroma

*Cystadenofibroma* is an uncommon benign epithelial ovarian tumour containing both epithelial and fibrous stromal components. This tumour may present as a purely cystic lesion, almost indistinguishable at MRI from a cystadenoma, although it more often shows a complex cystic appearance with thick septa and solid components. Foci of fibrous stroma have low signal intensity on T2-weighted images (Figs. 2 and 3) [29–31].

### Multilocular cystic masses

Multilocular cystic adnexal masses can be both benign or borderline and are represented by endometriomas, mucinous cystadenomas and tumours of borderline malignancy. Serous cystadenomas may rarely be multilocular.

*Endometriosis* is characterized by ectopic implantation of endometrial tissue outside the uterus, often involving the ovaries. Endometriotic cysts (or endometriomas or chocolate cysts) are often multifocal and bilateral, and usually appear as cystic to complex adnexal masses with high signal intensity on T1-weighted and intermediate to low signal intensity on T2-weighted images. Fat saturated T1-weighted sequences are helpful to rule out a fat-containing lesion and to confirm the presence of blood. On T2-weighted sequences, a gradual loss of signal intensity is called “shading sign” and is caused by repeated bleeding within the cyst. It has to be remembered that patients with endometriosis are at risk for developing ovarian malignancy (estimated risk about 2.5 %) [32]. Both endometrioid and clear cell tumours may be associated with endometriosis.

**Fig. 4:**
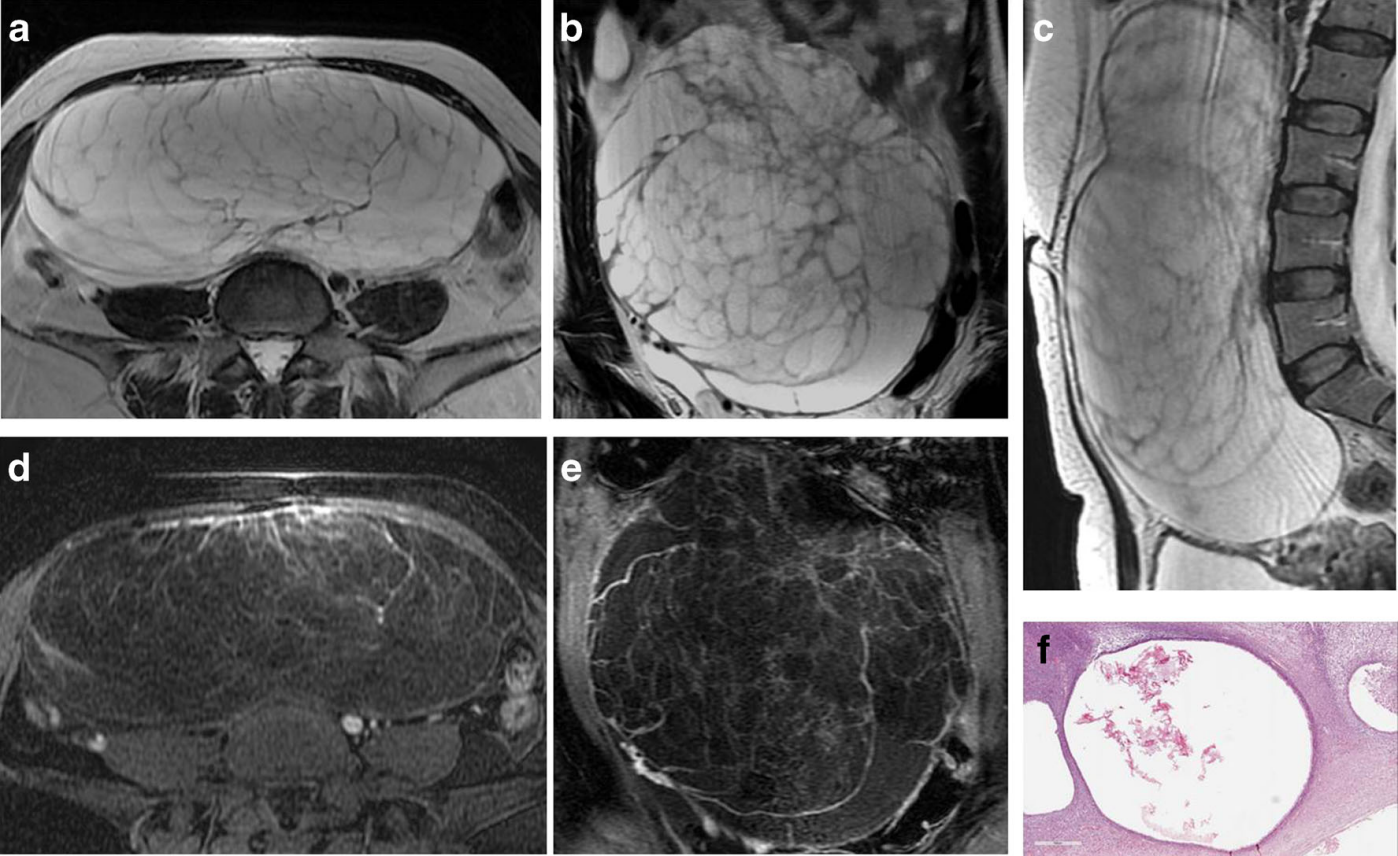
Mucinous cystadenoma in a 44-year-old woman. (**a**) Axial, (**b**) coronal and (**c**) sagittal T2-weighted images show a voluminous multilocular cystic mass with several septations. (**d**) Axial and (**e**) coronal contrast-enhanced fat-suppressed T1-weighted images demonstrate poor enhancement of the tumour wall and septa. (**f**) Photomicrograph (H&E X400) shows cystic spaces lined by columnar cells of intestinal type with mucinous secretion

*Mucinous cystadenoma* is a benign mucin-containing tumour, often larger than serous cystadenoma and monolateral rather than bilateral. It usually appears as a multilocular cystic lesion with a thin regular wall and several septations, lacking solid components and showing no significant enhancement after contrast administration (Fig. 4). The cystic loculi may have variable signal intensity on both T1-weighted and T2-weighted images, namely “stained glass appearance”, based on different mucin concentrations. It has to be remembered that pseudomyxoma peritonei can result from rupture of a mucinous cystadenoma.

**Fig. 5:**
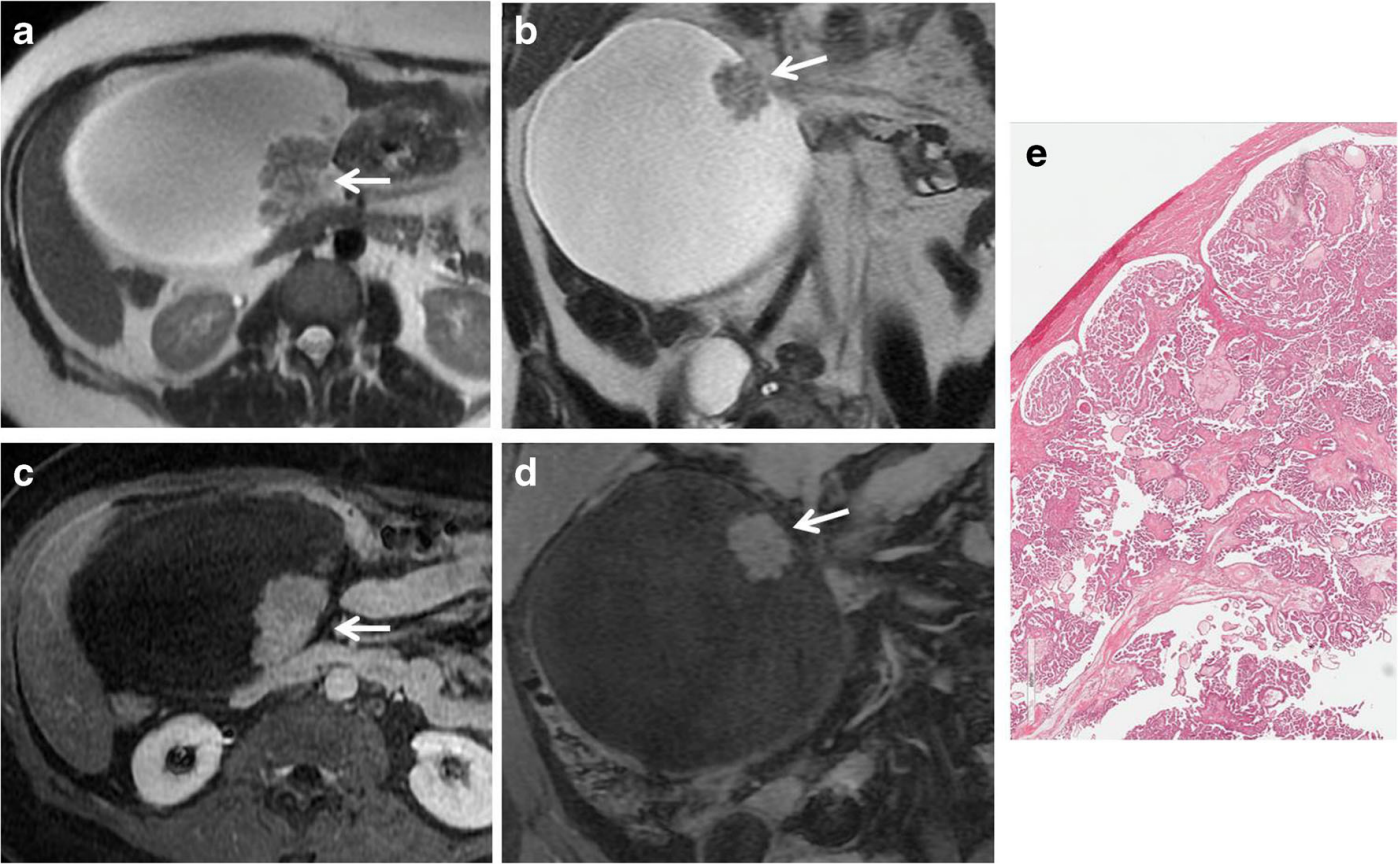
Borderline papillary-cystic serous tumour in a 43-year-old woman. (**a**) Axial and (**b**) coronal T2-weighted images show a cystic mass with papillary projections arising from the medial wall (*white arrows*). (**c**) Axial and (**d**) coronal contrast-enhanced fat-suppressed T1-weighted images show enhancement of the parietal solid component (*white arrows*). (**e**) Photomicrograph (H&E X30) shows intracystic papillary structures with fibrovascular axis

**Fig. 6:**
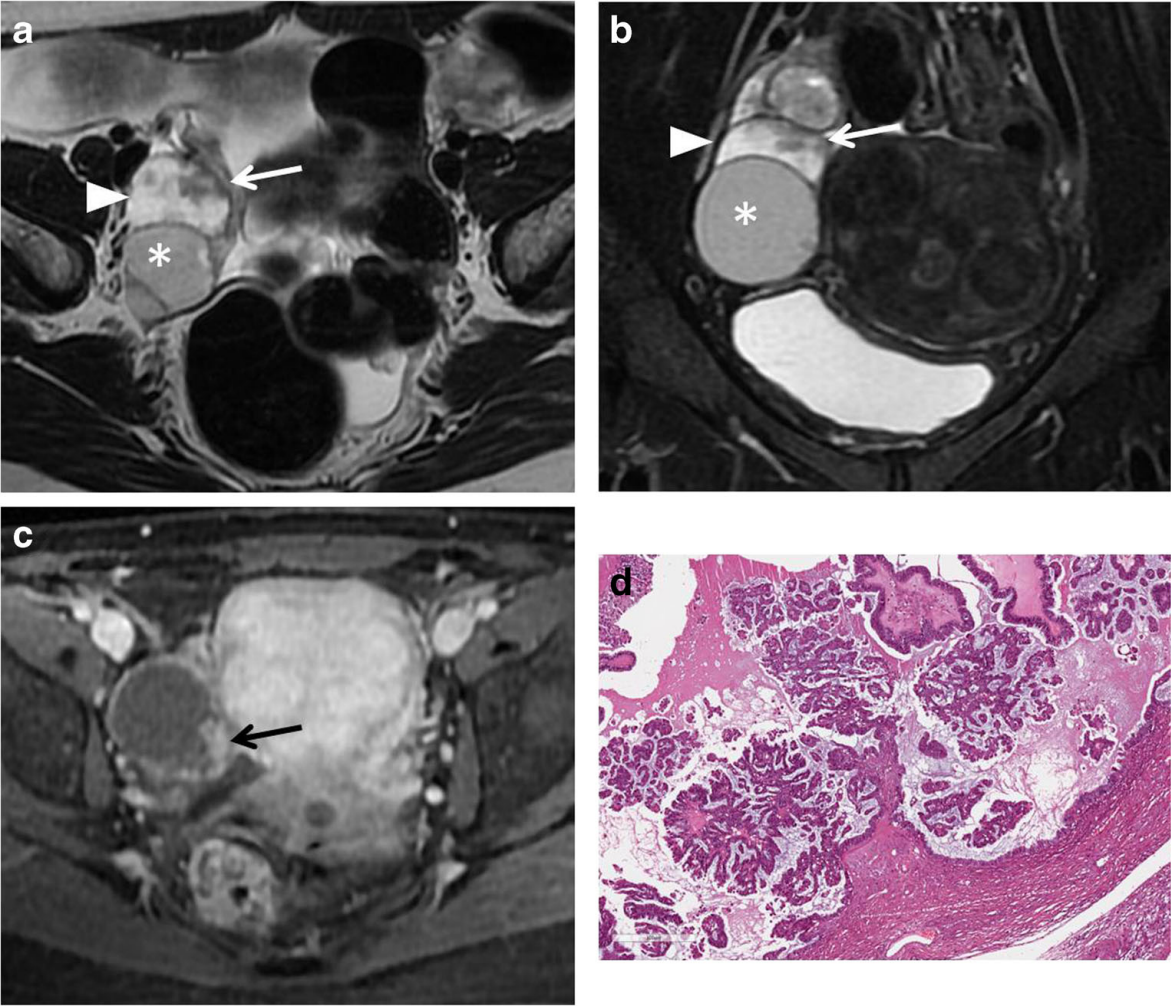
Borderline mucinous ovarian tumour in a 49-year-old woman. (**a**) Axial T2-weighted and (**b**) coronal fat-suppressed T2-weighted images show a multiloculated cystic ovarian tumour with intermediate (*) to high (*white arrowheads*) signal intensity of the loculi and solid parietal component (*white arrows*). (**c**) Axial contrast-enhanced fat-suppressed T1-weighted image demonstrates enhancement of the wall and of the solid component (*black arrow*). (**d**) Photomicrograph (H&E X300) shows neoplastic glands lined by columnar cells of intestinal-type with mucinous secretion

*Borderline tumours* are a group of ovarian neoplasms histologically characterized by epithelial anaplasia [33]. Compared to malignant ovarian cancer, they occur in younger patients. Serous and mucinous cystadenoma account for most borderline tumours; however, other histological types, such as endometrioid and clear cell tumours, are sometimes seen. These tumours usually show non-invasive behaviour, but it is also possible for them to present with lymph node involvement and peritoneal implants; however, they are associated with a better prognosis than cystoadenocarcinomas. The morphological appearance of borderline tumours is between benign and malignant ones. Borderline serous cystadenoma usually manifest as a complex cystic lesion with some septa and papillary projections (Fig. 5). Both benign and borderline mucinous cystadenomas have a multilocular appearance, and it is impossible to make a differential diagnosis at MRI (Fig. 6).

### Cystic and solid masses

A mixed cystic and solid appearance of an ovarian mass should raise a suspicion of malignancy, like surface epithelial tumours and metastasic lesions. However, a benign lesion like mature cystic teratoma (or ovarian dermoid cyst) also appears as a complex mass. A germ cell tumour (Yolk sac tumour) and sex cord-stromal tumours (granulosa cell) are also described in this section.

**Fig. 7:**
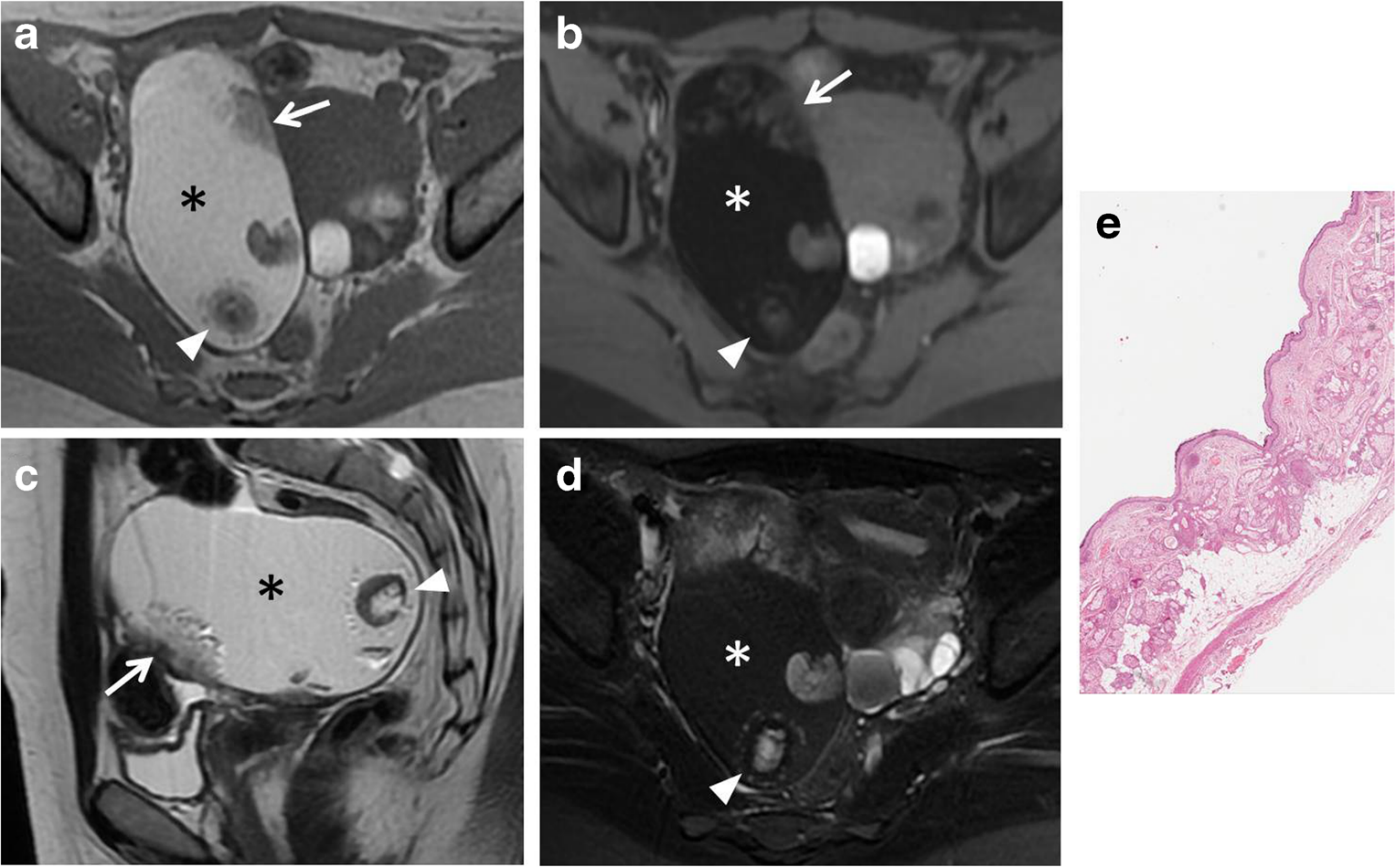
Mature teratoma in a 33-year-old woman. (**a**) Axial T1-weighted, (**b**) axial fat-suppressed T1-weighted, (**c**) sagittal T2-weighted and (**d**) axial fat-suppressed T2-weighted images show a well-defined ovalar mass with fat tissue (*) with high signal intensity both on T1-weighted and T2-weighted images and signal loss on fat-suppressed sequences. A hypointense nodule (*white arrowheads*) is seen within the lesion, as well as a solid component (*white arrows*). (**e**) Photomicrograph (H&E X20) shows cystic space lined by mature epidermis. Skin appendages and neural tissue are seen in the wall

**Fig. 8:**
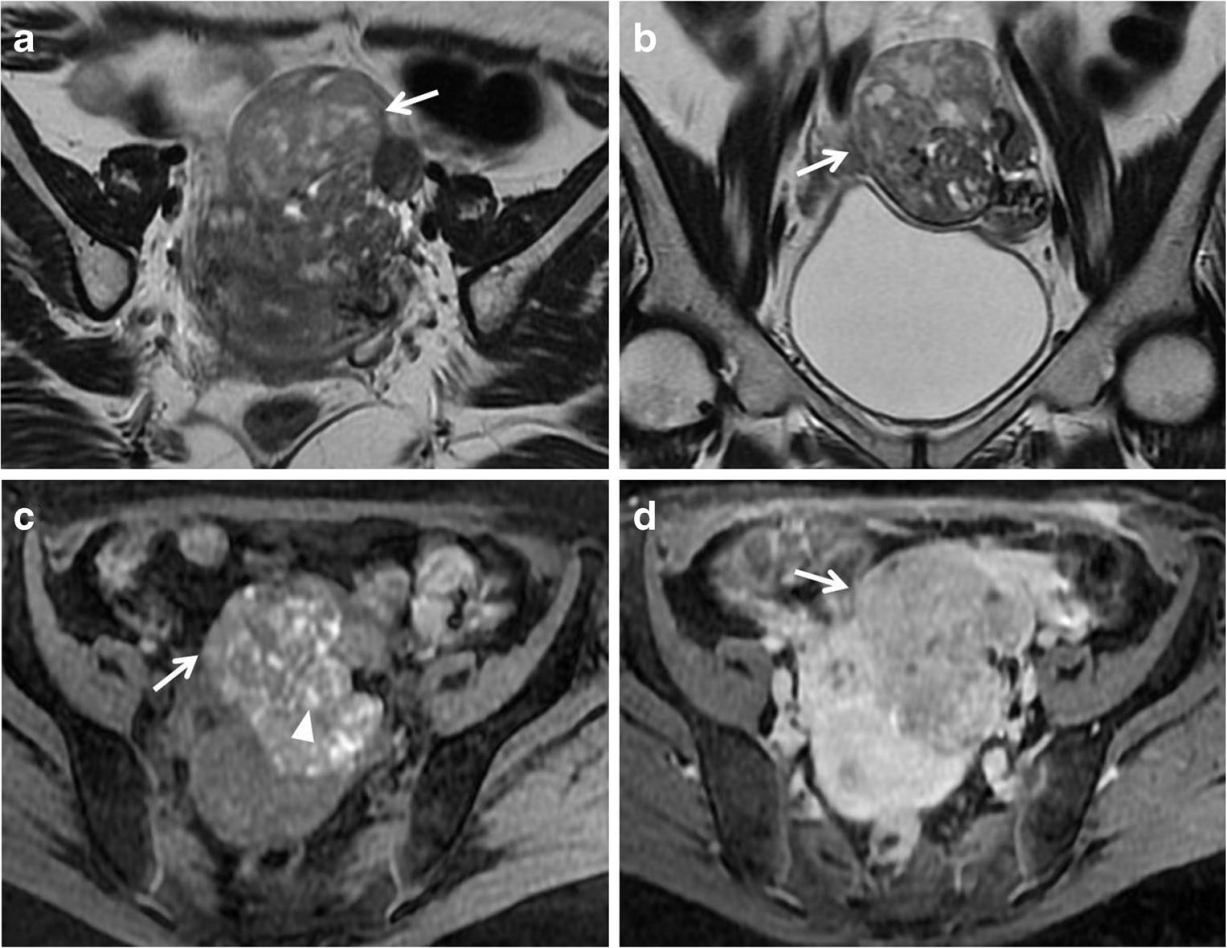
Struma ovarii in a 32-year-old woman. (**a**) Axial T2-weighted, (**b**) coronal T2-weighted and (**c**) axial fat-suppressed T1-weighted images show a predominantly solid mass (*white arrows*) with multiple cystic spaces of variable signal intensity, some of which show high signal intensity on T1-weighted image (*white arrowhead*). (**d**) Axial contrast-enhanced fat-suppressed T1-weighted image demonstrates enhancement of the mass (*white arrow*)

*Mature cystic teratoma* is the most common ovarian neoplasm and affects mostly young patients. It is a benign germ cell tumour consisting of at least two of the three embryogenic germ cell layers, and usually contains ectodermal (skin, brain), mesodermal (fat, bone) and/or endodermal (thyroid tissue, gastrointestinal and bronchial epithelium) mature tissue [34]. Simultaneous presence of these components leads to a complex and heterogeneous appearance; however, the key to a correct diagnosis is detection of fat-tissue within the mass. MRI usually demonstrates tissue with high signal intensity both on T1-weighted and T2-weighted images and signal loss on fat suppression sequences (Fig. 7). A T1-weighted fat-saturated sequence is also able to make differential diagnosis between fat-tissue and haemorrhagic lesions, namely endometrial cysts. A fat-fluid interface may also be found, and is typical of mature cystic teratoma. Other features are areas of low signal intensity (teeth), soft-tissue protuberances (Rokitansky nodules) and floating debris [35]. Malignant transformation of mature cystic teratomas is rare (1–2 % of cases), and usually occurs in postmenopausal women due to a squamous cell carcinoma arising from the cyst wall. In addition, it has to be known that there are other less common types of ovarian teratomas, such as the malignant immature teratoma, which may have less cystic elements or be completely solid, and the benign monodermal teratoma, in which one of the three germ cell layers is predominant. *Struma ovarii* is one of the main subtypes of ovarian monodermal teratomas (approximately 3 % of all mature teratomas). It is mainly composed of thyroid tissue; thus, it may be associated with symptoms or signs of thyrotoxicosis. MRI demonstrates a complex mass with multiple cystic and solid areas. Cystic spaces may show variable signal intensity demonstrating both high and low signal intensity on T1-weighted and T2-weighted images; in particular, some cystic spaces demonstrate low signal intensity on both T1-weighted and T2-weighted images because of the colloid of the struma (Fig. 8) [36, 37]. No fat-tissue can be found in this lesion.

**Fig. 9:**
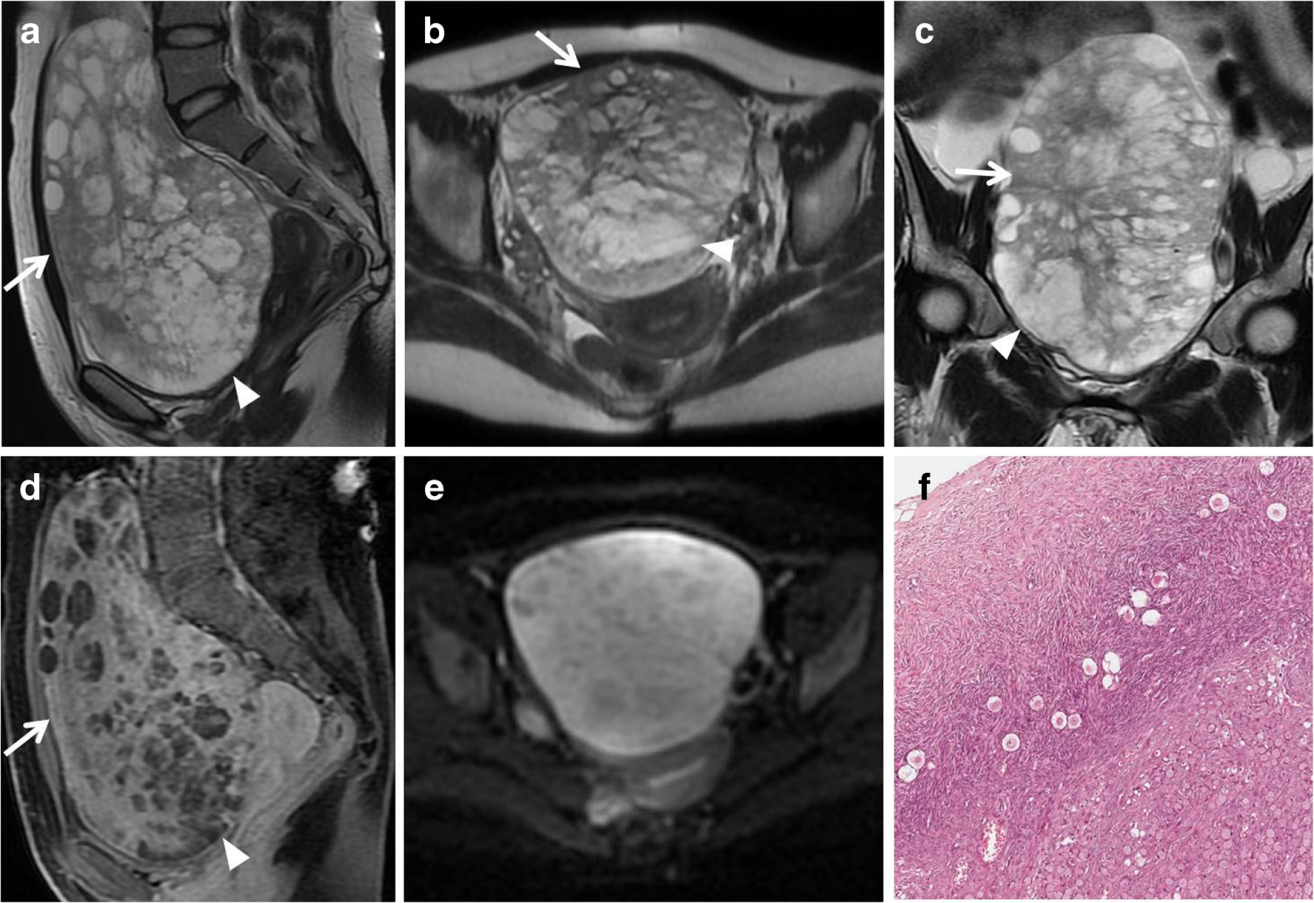
Krukenberg tumour from gastric carcinoma in a 23-year-old woman. (**a**) Sagittal, (**b**) axial and (**c**) coronal T2-weighted images show a heterogeneous mass characterized by solid components with low signal intensity (*white arrows*) and cystic components with high signal intensity (*white arrowheads*). (**d**) Sagittal contrast-enhanced fat-suppressed T1-weighted image demonstrates enhancement of the solid components (*white arrow*). (**e**) Axial DW image (b = 800 s/mm^2^) demonstrates increased signal of the solid components of the lesion indicating hypercellularity. (**f**) Photomicrograph (H&E X200) shows ovarian parenchyma diffusely infiltrated by signet-ring cells

*Ovarian metastasis* account for about 5 % of malignant ovarian tumours, and there is a potential risk of misjudgment if the primary tumour is not known. Stomach, colon, breast, lung and contralateral ovary are the most frequent neoplasms to metastasize to the ovaries. These tumours can spread to the ovaries by direct invasion, transcoelomic dissemination, hematogeneous and lymphatic spread. Krukenberg tumours are defined as metastatic lesions to the ovary from a gastrointestinal cancer and characterized by mucin-producing signet-ring cells (Fig. 9). Ovarian metastasis are more often bilateral with a cystic and solid or a predominant solid morphological appearance. An important enhancement of the cystic wall and of the solid component is usually seen after gadolinium administration. It is very difficult to distinguish between primary and secondary ovarian malignancy on diagnostic imaging, mostly because of the absence of typical features helping the differential diagnosis [38].

*Surface epithelial-stromal tumours* account for 65 % of all ovarian tumours and represent 85 % of ovarian malignancies. In this section, we describe serous cystadenocarcinoma, mucinous cystadenocarcinoma, endometrioid tumours and clear cell tumours, whereas transitional cell (Brenner) tumours will be described in the next one because of their predominantly solid appearance.

**Fig. 10:**
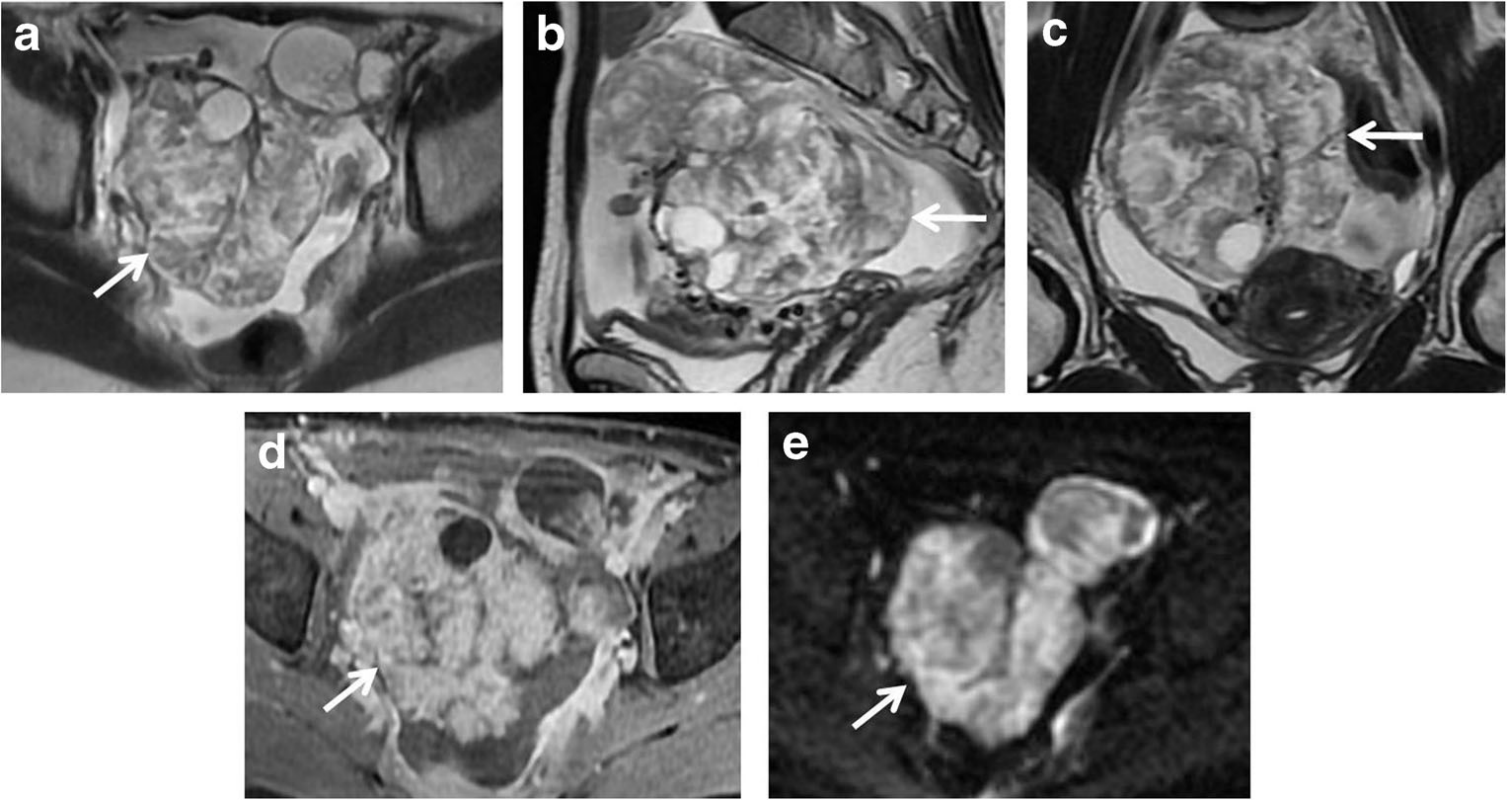
Serous papillary carcinoma in a 48-year-old woman. (**a**) Axial, (**b**) sagittal and (**c**) coronal T2-weighted image show a cystic and solid mass (*white arrows*). (**d**) Axial contrast-enhanced fat-suppressed T1-weighted image shows the enhancing exophytic papillary projections of the tumour (*white arrow*). (**e**) Axial DW image (*b* = 800 s/mm^2^) demonstrates increased signal of the lesion (*white arrow*), indicating hypercellularity

**Fig. 11:**
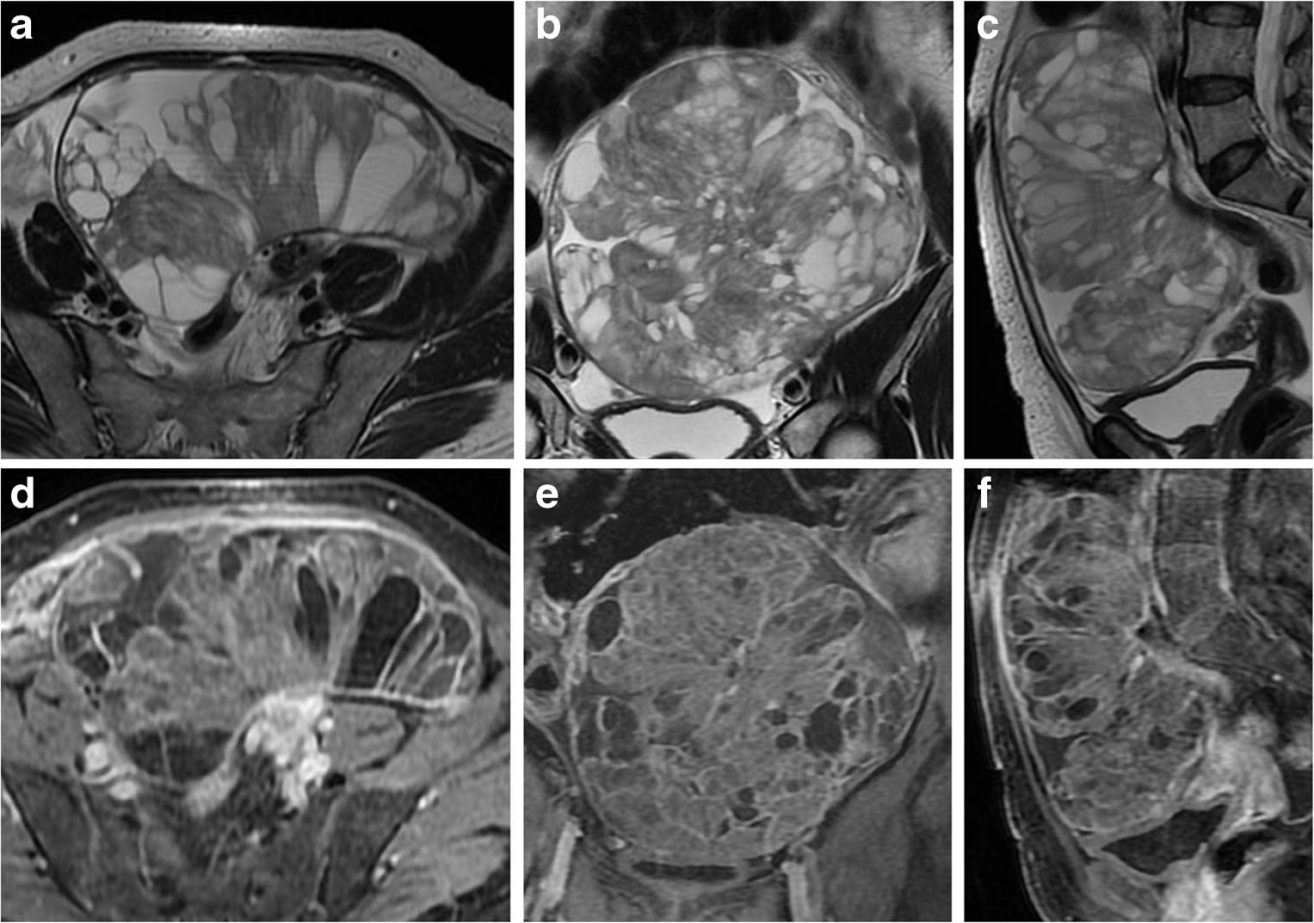
Mucinous cystadenocarcinoma in a 57-year-old woman. **(a)** Axial, **(b)** coronal and **(c)** sagittal T2-weighted images show a large mass with mixed solid and multilocular cystic appearance with low signal intensity of the solid component and variable signal intensity within the locules (“stained glass appearance”). **(d)** Axial, **(e)** coronal and **(f)** sagittal contrast-enhanced fat-suppressed T1-weighted images demonstrate marked enhancement of the solid component, wall and septa of the tumour

**Fig. 12:**
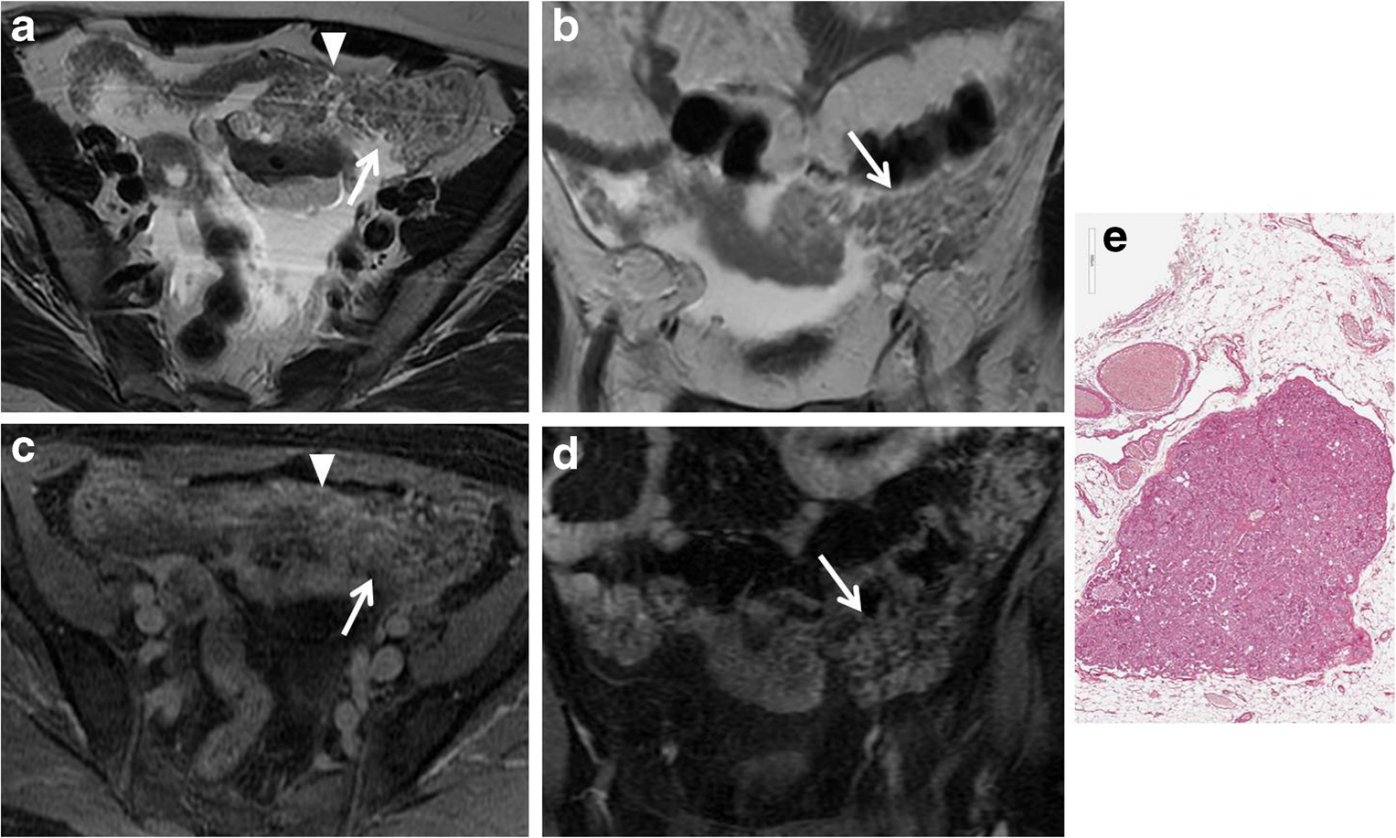
Peritoneal carcinomatosis due to disseminated ovarian papillary serous cystadenocarcinoma in a 63-year-old woman. (**a**) Axial T2-weighted, (**b**) coronal T2-weighted, (**c**) axial T1-weighted, (**d**) coronal T1-weighted images show multiple tumour implants in the left paracolic gutter (*white arrows*) and omental implants (*white arrowheads* in **a**, **c**). (**e**) Photomicrograph (H&E X200) shows solid and papillary structures invading omental adipose tissue

*Serous cystadenocarcinoma* is the most common, and is responsible for about 40 % of malignant ovarian neoplasms, whereas *mucinous cystadenocarcinoma* is less common and accounts for about 10 % of ovarian malignancies. These tumours are seen as complex multilocular masses, usually with thick and irregular walls, septations, solid components and papillary projections of low signal intensity on T2-weighted images with contrast enhancement after gadolinium administration (Figs. 10 and 11). The serous fluid of the cystic components has low to intermediate signal intensity on T1-weighted and high signal intensity on T2-weighted images. In some cases of serous cystadenocarcinoma there are psammomatos calcifications, better seen on CT scan because of their high attenuation. Signal intensity of mucinous content is variable depending on mucin concentration. These tumours can be very large, even greater than 12–15 cm. Serous cystadenocarcinoma is more frequently bilateral than mucinous cystadenocarcinoma. They may be responsible for peritoneal invasion leading to peritoneal carcinomatosis (Fig. 12).

**Fig. 13:**
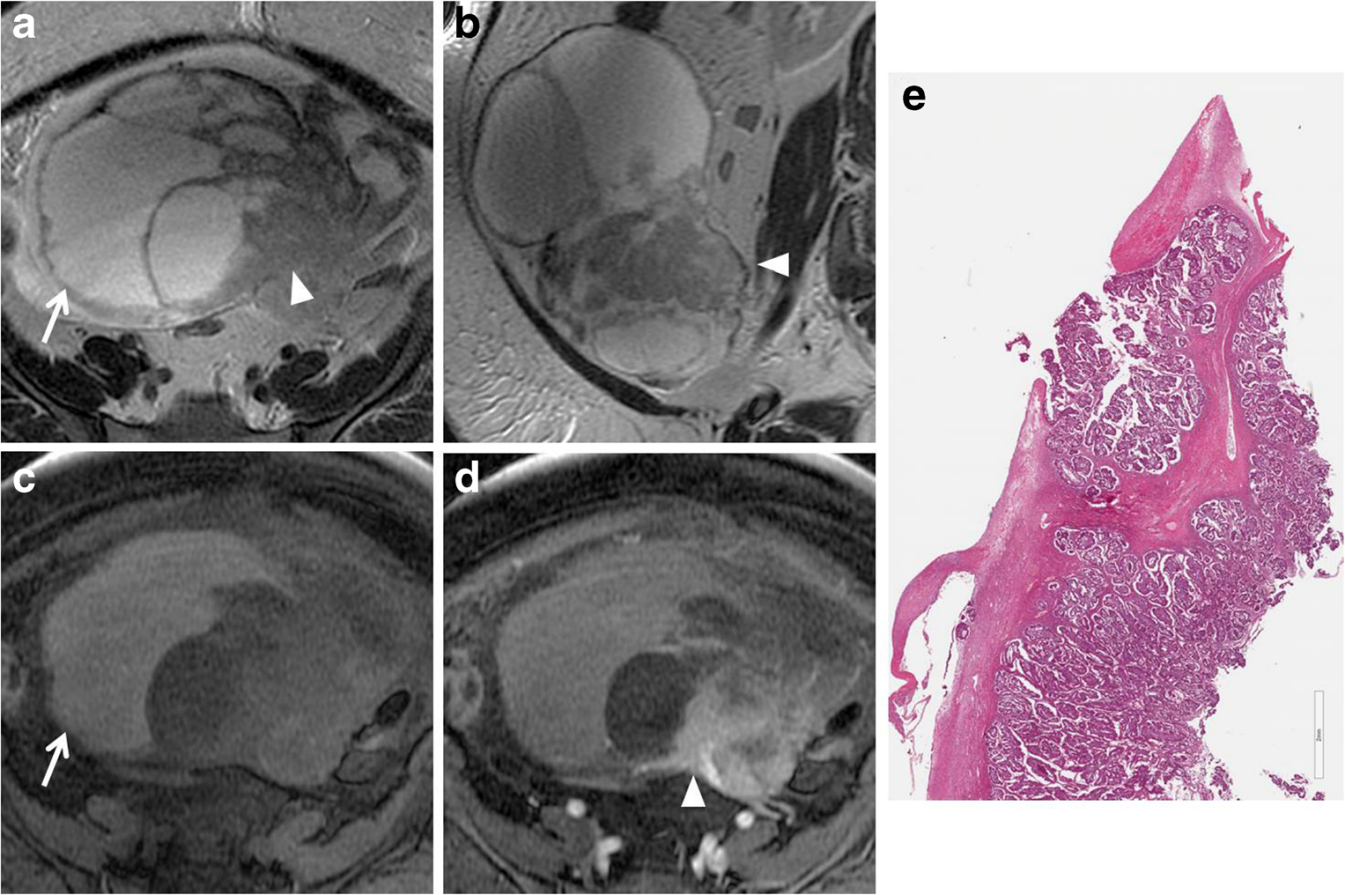
Endometrioid adenocarcinoma in a 57-year-old woman. (**a**) Axial and (**b**) coronal T2-weighted images show complex masses with solid (*white arrowheads*) and cystic (*white arrows*) components. On (**c**) axial fat-suppressed T1-weighted image, the cystic component shows haemorrhagic hyperintense signal (*white arrow*). On (**d**) axial contrast-enhanced fat-suppressed T1-weighted image, the solid component shows marked enhancement (*white arrowhead*). (**e**) Photomicrograph (H&E X20) shows capsular invasion by neoplastic glands

**Fig. 14:**
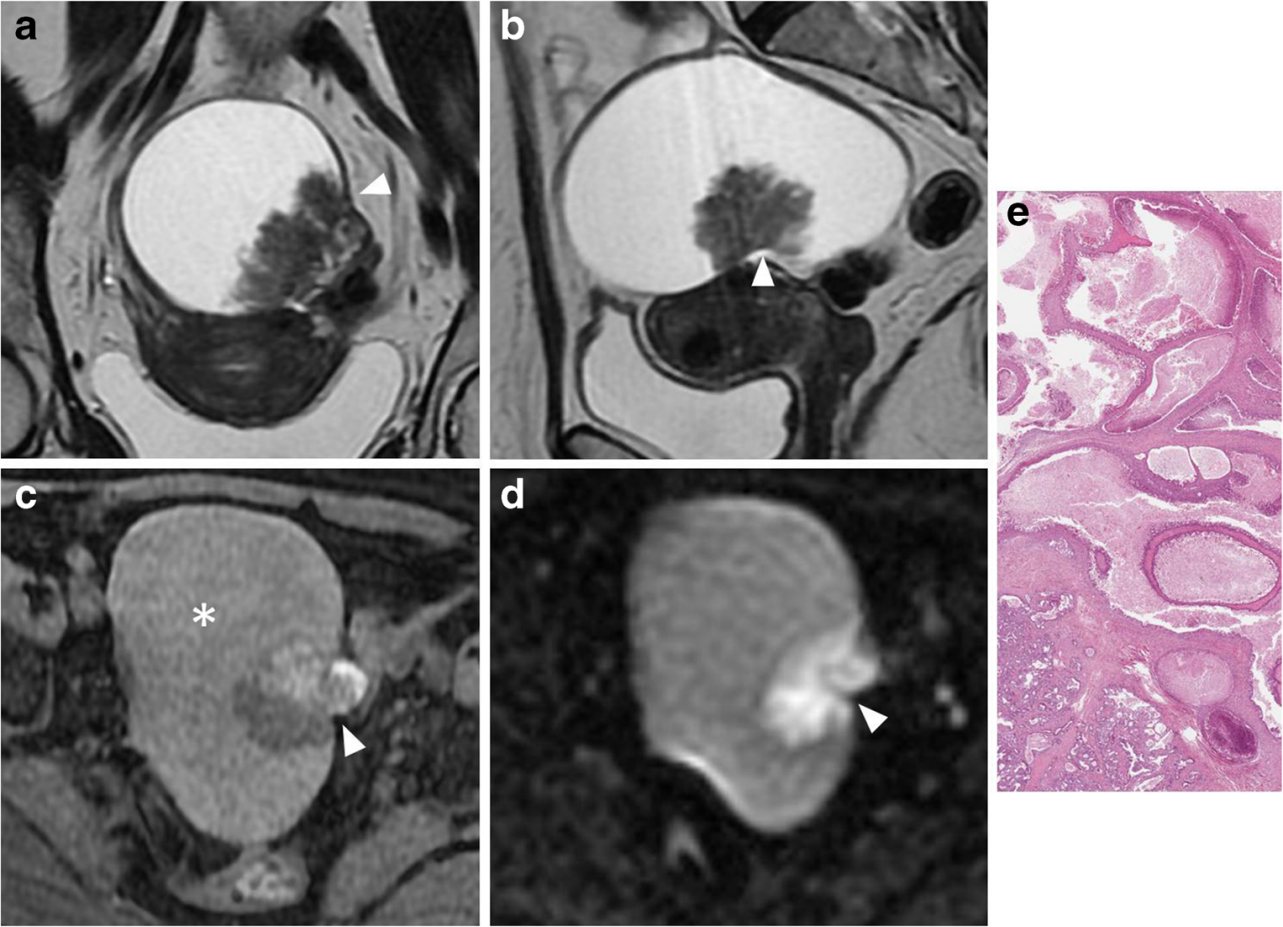
Endometrioid adenocarcinoma in a 60-year-old woman. (**a**) Coronal and (**b**) sagittal T2-weighted images show a predominantly cystic mass with solid component arising from the inferior wall (*white arrowheads*). (**c**) Axial fat-suppressed T1-weighted image shows hyperintense haemorrhagic content of the cyst (*) and solid parietal component (*white arrowhead*). (**d**) Axial DW image (*b* = 800 s/mm^2^) demonstrates increased signal of the solid component of the lesion (*white arrowhead*) indicating hypercellularity. (**e**) Photomicrograph (H&E X300) shows neoplastic glands cystically dilated

*Endometrioid and clear cell tumours* are commonly associated with endometriosis. These tumours usually appear as complex masses with solid and cystic components, although they can also be predominantly cystic (Figs. 13 and 14) [39]. However, the rapid growth of an endometrioma, along with multilocularity and presence of mural nodules within the haemorrhagic cyst with enhancement after gadolinium administration should raise the suspicion of malignancy [32, 40].

**Fig. 15:**
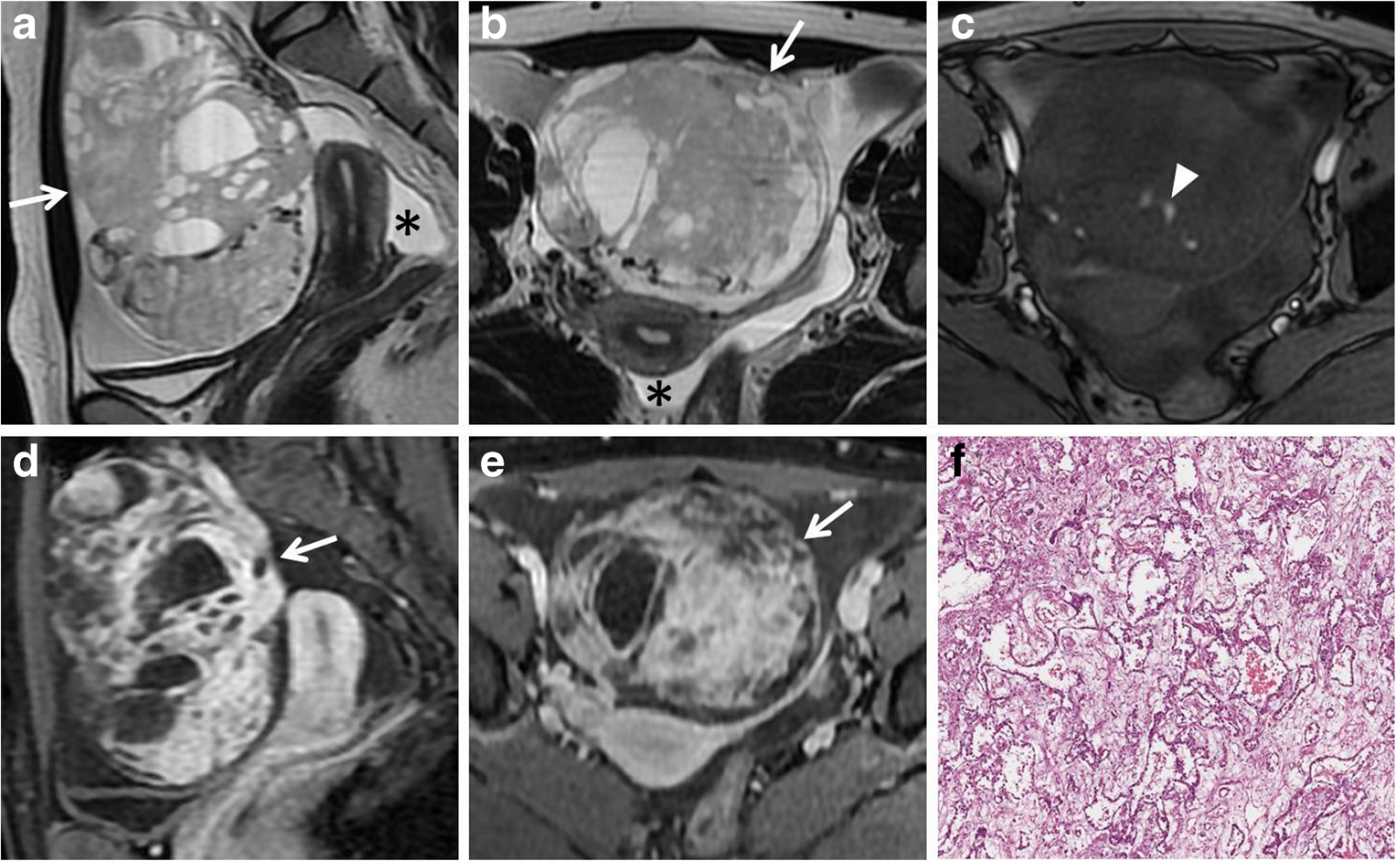
Yolk sac tumour in a 26-year-old woman. (**a**) Sagittal and (**b**) axial T2-weighted images show a mixed cystic and solid mass (*white arrows*). (**c**) Axial T1-weighted image shows haemorrhagic areas with high signal intensity within the lesion (*white arrowhead*). (**d**) Sagittal and (**e**) axial contrast-enhanced fat-suppressed T1-weighted images show marked enhancement of the solid component (*white arrows*). Peritoneal fluid can also be seen (* in **a**, **b**). (**f**) Photomicrograph (H&E X200) shows endodermal sinus pattern with Schiller-Duval bodies; these structures are covered by tumour cells and has a central capillary

*Yolk sac tumour*, also known as endodermal sinus tumour, is relatively rare and is responsible for about 1 % of ovarian malignancies. This tumour generally presents in the second or third decade as a large mixed cystic and solid mass with the bright dot sign, namely foci of enhancement due to dilated vessels (small aneurisms) as a result of increased vascularity [41]. It is not uncommon to find haemorrhagic areas with high signal intensity on T1-weighted images (Fig. 15).

*Granulosa cell tumours* are usually benign; however, they may be malignant as well. Clinically, they can manifest by producing hormones with hyperestrogenism. Granulosa cell tumours can be divided into two main subtypes, adult and juvenile. The adult type is responsible for about 95 % of cases and occurs preferentially in perimenopausal and postmenopausal women, resulting in endometrial hyperplasia and atypical bleeding. The juvenile type is less frequent and affects prepuberal children, leading to pseudoprecocity. Granulosa cell tumours appear mostly as both cystic and solid masses, but they can also have a multilocular cystic or a predominantly solid appearance [42, 43].

### Predominantly solid masses

Predominantly solid adnexal masses account for lesions with benign, borderline and malignant behaviour. They include tumours of different origin: epithelial (transitional cell – Brenner tumour and, rarely, serous and mucinous carcinomas), germ cell (dysgerminoma and Yolk sac tumour), sex cord (fibrothecomas, granulosa cell tumours and Sertoli-Leydig cell tumours) and metastatic lesions.

*Brenner tumour* is responsible for about 2 % of ovarian neoplasms; it is also named “transitional cell tumour” because of its histological similarity to the urotelium. It is more often benign. On T2-weighted images, Brenner tumour shows a low signal intensity because of its solid fibrous content. In some cases, areas of calcification within the solid components can be found, best demonstrated on both US and CT, rather than MRI. Brenner tumour can be associated with other cystic tumours, like mucinous cystadenomas, so that MRI demonstrates a lesion with both cystic (cystadenomas) and solid (Brenner) components [44]. Although rarely, both borderline and malignant lesions (transitional cell carcinomas) exist with complex cystic and solid appearances.

*Dysgerminoma* usually appears as a solid lobulated lesion with fibrovascular septa, surrounded by a fibrotic capsule. The solid component shows intermediate to high signal intensity on T2-weighted images, whereas the septa appear at low signal intensity, but show a significant contrast enhancement [41].

**Fig. 16:**
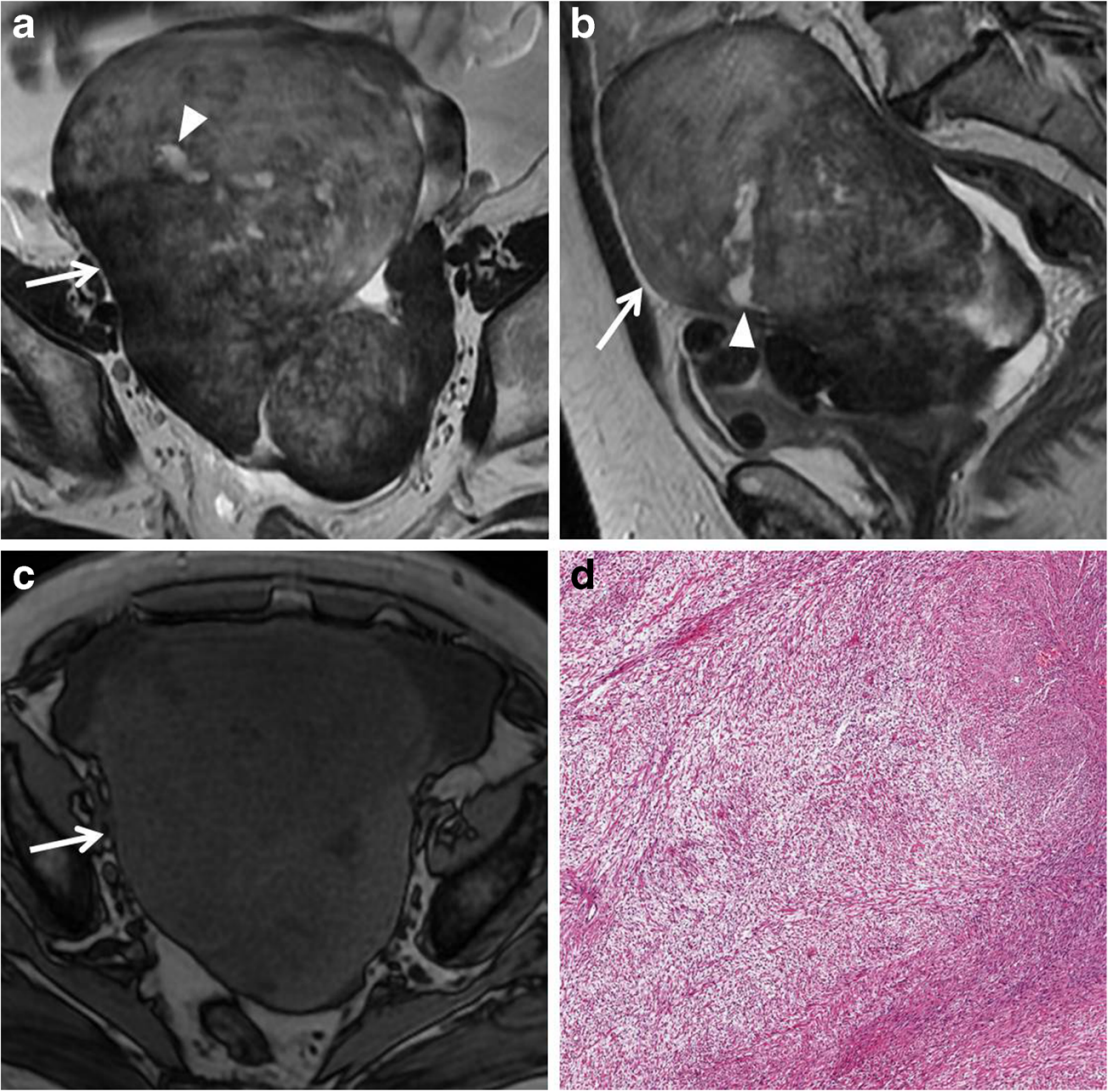
Fibrothecoma in a 66-year-old woman. (**a**) Axial and (**b**) sagittal T2-weighted images show a heterogeneous solid mass (*white arrows*) with low to intermediate signal intensity. Some areas of cystic degeneration with high signal intensity can be seen within the lesion (*white arrowheads*). (**c**) Axial T1-weighted image confirms the solid appearance of the lesion (*white arrow*). (**d**) Photomicrograph (H&E X200) shows fascicles of spindle cells with centrally placed nuclei and a moderate amount of pale cytoplasm

**Fig. 17:**
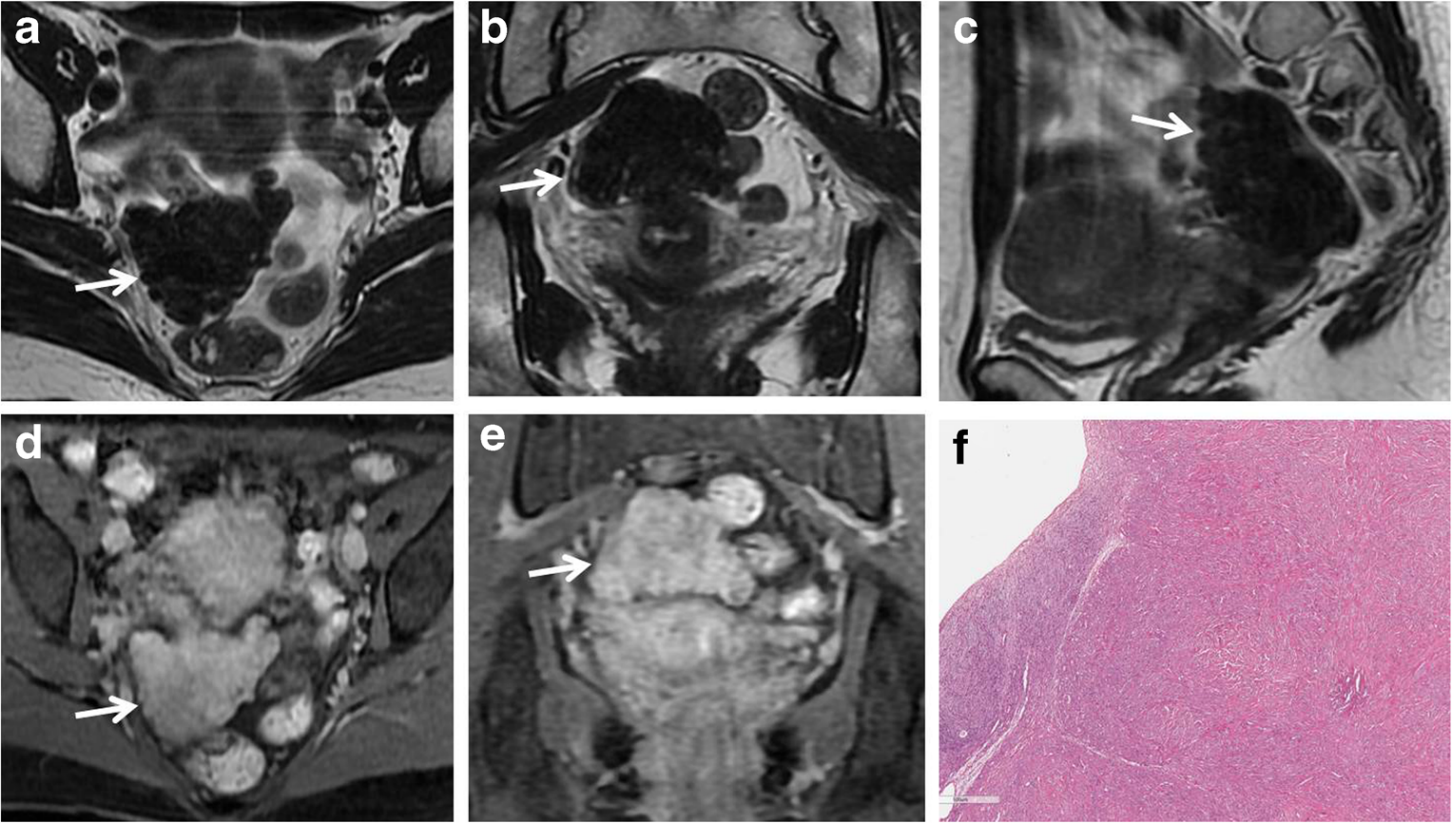
Fibroma in a 44-year-old woman. (**a**) Axial, (**b**) coronal and (**c**) sagittal T2-weighted images show a polilobulated solid mass (*white arrows*) with homogeneous low signal intensity. (**d**) Axial and (**e**) coronal contrast-enhanced fat-suppressed T1-weighted images show the homogeneous enhancement of the lesion (*white arrows*). (**f**) Photomicrograph (H&E X20) shows closely packed spindle stromal cells arranged in storiform pattern with hyaline bands

*Fibromas, thecomas *and *fibrothecomas* are classified as sex cord-stromal tumours and are the most common benign solid lesion of the ovaries. These tumours are usually asymptomatic and there may be an association with ascites and pleural effusion (Meig syndrome). *Fibrothecomas* consist of both fibrous tissue and theca cells with lipidic content. They present as a well-circumscribed solid mass with low to intermediate signal intensity on T1-weighted and low signal intensity on T2-weighted images, although there may be some scattered areas of cystic degeneration with high signal intensity on T2-weighted scans (Fig. 16). In *fibromas*, the prominent fibrosis with abundant collagen content is responsible for the low signal intensity (Fig. 17), while in *thecomas*, the mainly lipidic content of theca cells may be depicted at chemical-shift imaging [7]. After contrast administration, only a minimal enhancement can be demonstrated. Differential diagnosis of fibrothecomas includes pedunculated uterine leiomyomas; however, the multiplanar imaging along with the absence of the normal ipsilateral ovary are useful in distinguishing them.

*Sclerosing stromal tumour* is classified as sex cord-stromal tumours into the thecoma-fibroma group. It affects mostly young women, manifesting as menstrual irregularities. This tumour presents as predominantly solid mass; after contrast administration, it shows an early peripheral enhancement with centripetal progression [43, 45, 46].

**Fig. 18:**
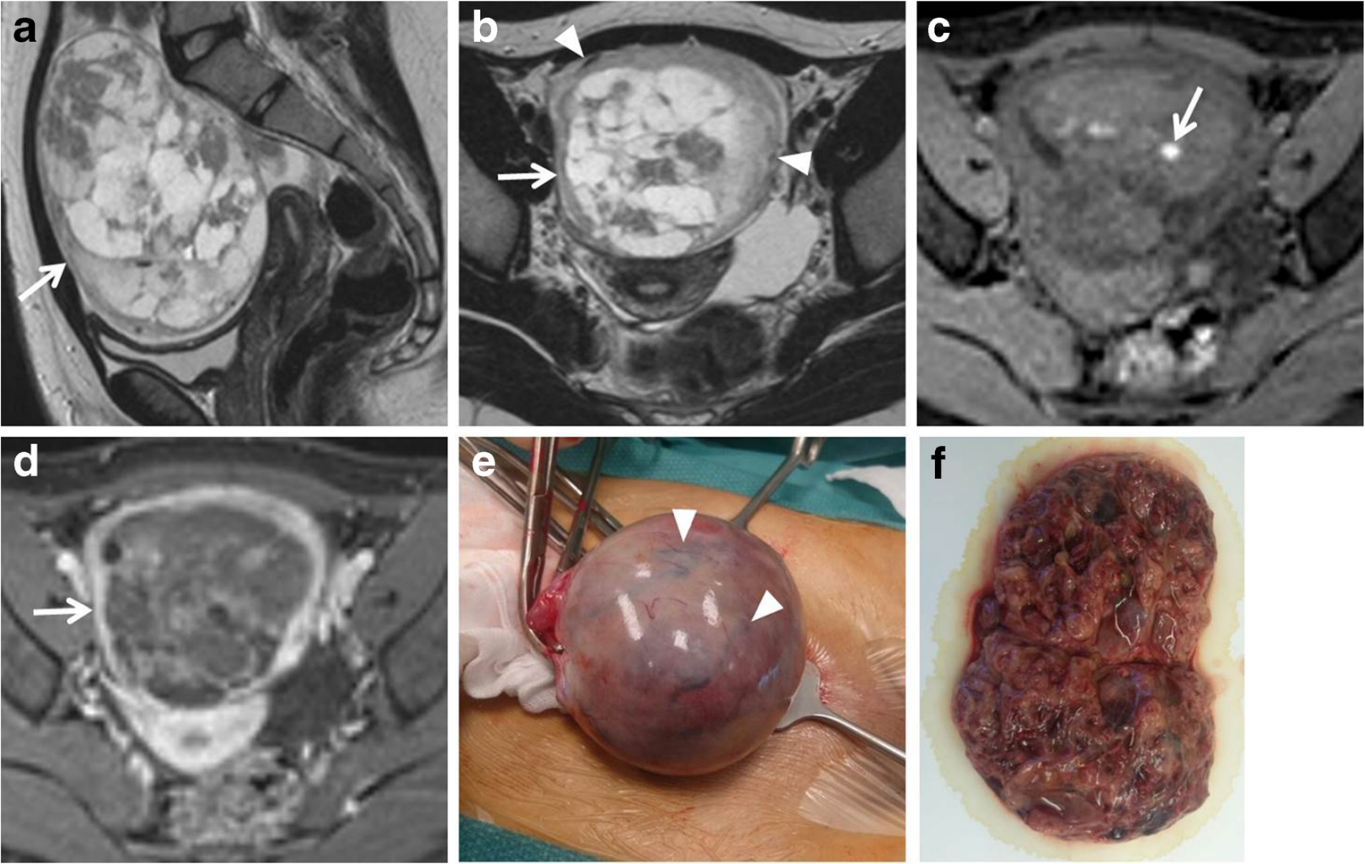
Sertoli-Leydig cell tumour in a 13-year-old woman. (**a**) Sagittal and (**b**) axial T2-weighted images show a voluminous mixed cystic and solid mass (*white arrows*). At the periphery of the mass, some signal flow-voids that represent vascular structures are recognizable (*white arrowheads*). (**c**) An Axial T1-weighted image shows haemorrhagic areas with high signal intensity within the lesion (*white arrow*). (**d**) Axial contrast-enhanced fat-suppressed T1-weighted image shows enhancement of the solid component, more evident in the peripheral portion (*white arrow*). (**e**) Photograph of the operating field shows the mass with vascular structures on its surface (*white arrowheads*). (**f**) Photograph of the cut surface of the resected lesion shows good MR imaging-histopathologic correlation of the mass

*Sertoli-Leydig cell tumours* account for only 0.5 % of ovarian tumours; however, they can be responsible for hyperandrogenism and are the most common virilizing tumours. These tumours usually affect young women and signs of androgen activity can be seen in about one-third of patients. Sertoli-Leydig cell tumours usually appear as solid masses; however, they may also present as heterogeneous lobulated lesions with both cystic and solid components [41, 47]. On T2-weighted images, most of these tumours show a predominantly low signal intensity of the solid components, relating to the fibrous stroma, with some scattered cystic areas of high signal intensity (Fig. 18) [46, 48].

## Conclusion

Morphological characteristics of adnexal masses range from cystic (both unilocular and multilocular), complex (cystic and solid) and predominantly solid. It is true that there is a certain overlap between benign and malignant tumours; in particular, it has to be considered that borderline lesions are very difficult to differentiate because they have both benign and malignant features.

In 2007, Sohaib et al. showed that from the analysis of the MR imaging features, “the most predictive characteristics of malignancy are vegetations in a cystic lesion, presence of ascites, a maximal diameter greater than 6 cm, and necrosis in a solid lesion” [33]. In 2011, Valentini et al. suggested criteria for characterization of benign and suspicious adnexal lesions, indicating as features suggestive of malignancy the demonstration of “solid, solid/cystic enhancing masses (greater than 4 cm in maximum diameter) with papillary projections and irregular thick wall and septa (greater than 3 mm) into a cystic lesion” as well as a “heterogeneous and early enhancement pattern” [35].

To conclude this work, we’d like to indicate some key imaging features and general advice that could be of help in the differential diagnosis of adnexal masses:

- non-neoplastic lesions should always be taken into consideration;
- in a patient with endometriosis, the presence of a complex and rapidly growing mass with contrast enhancement should raise the suspicion of endometrioid or clear cell tumour;
- a very low signal intensity on T2-weighted images indicates a fibrotic component, suggesting a tumour of the thecoma/fibroma group, a cystadenofibroma or a Brenner tumour;
- sequences with fat saturation are helpful when facing a mass with high signal intensity on T1-weighted images, because the signal suppression is suggestive of a teratoma; otherwise, an endometrial cysts or other haemorrhagic lesions should be considered;
- a “stained glass appearance” with cystic loculi of variable signal intensity usually refers to a mucinous tumour, because of the different mucin concentration;
- metastasis should be considered if a complex enhancing mass is demonstrated in both ovaries (eventually looking for an unknown primary tumour); however, it must be remembered that serous epithelial tumours can be bilateral as well.
